# The feline cutaneous and oral microbiota are influenced by breed and environment

**DOI:** 10.1371/journal.pone.0220463

**Published:** 2019-07-30

**Authors:** Caitlin E. Older, Alison B. Diesel, Sara D. Lawhon, Cintia R. R. Queiroz, Luan C. Henker, Aline Rodrigues Hoffmann

**Affiliations:** 1 Department of Veterinary Pathobiology, College of Veterinary Medicine & Biomedical Sciences, Texas A&M University, College Station, TX, United States of America; 2 Department of Small Animal Clinical Sciences, College of Veterinary Medicine & Biomedical Sciences, Texas A&M University, College Station, TX, United States of America; Skin Research Institute Singapore, SINGAPORE

## Abstract

Previous research revealed the feline skin bacterial microbiota to be site-specific and the fungal microbiota to be individual-specific. The effect of other factors, such as genotype and environment, have not yet been studied in cats, but have been shown to be potentially important in shaping the cutaneous microbiota of other animals. Therefore, the objectives of this study were to evaluate the effect of these factors on the bacterial and fungal microbiota of feline skin and oral cavity. The influence of genotype was assessed through the analysis of different cat breeds, and the influence of environment through comparison of indoor and outdoor cats. DNA was extracted from skin and oral swabs, and bacterial and fungal next-generation sequencing were performed. Analysis of the skin microbiota of different cat breeds revealed significant differences in alpha diversity, with Sphynx and Bengal cats having the most diverse communities. Many taxa were found to be differentially abundant between cat breeds, including *Veillonellaceae* and *Malassezia* spp. Outdoor environment exposure had considerable influence on beta diversity, especially in the oral cavity, and resulted in numerous differentially abundant taxa. Our findings indicate that the oral bacterial microbiota and both fungal and bacterial microbiota of feline skin are influenced by breed, and to a lesser degree, environment.

## Introduction

Until recently, the feline skin microbiota had not been described using next-generation sequencing. We now know that feline skin is inhabited by bacterial communities that are distinct to each body site[1] and fungal communities more unique to the individual cat.[2] Additionally, the composition of the feline cutaneous microbiota is more diverse than previously described in culture-based studies.[3] Like canine[4–6] and human[7–10] skin, the primary bacterial phyla present on cats are Proteobacteria, Firmicutes, Actinobacteria, and Bacteroidetes, although in different proportions. Unlike human skin which is primarily colonized by *Malassezia* spp.,[10–12] canine[13] and feline[2] skin are colonized by a more diverse fungal mycobiota, with Dothideomycetes (mainly *Cladosporium* spp., *Alternaria* spp., *Epicoccum* spp.), a class of many environmental fungi, being the predominant one found.

The feline oral cavity also has a diverse and unique microbiota. Due to the prevalence of oral disease[14–18] and cat bite infection,[19–21] which are known to be associated with bacteria, much of the feline oral microbiota researched has focused on the bacterial populations present; however the fungal oral microbiota has been described in a previous study focused on the cutaneous microbiota.[2] The feline oral bacterial communities are similar to what has been described on the skin, but with increased abundances of Bacteroidetes,[1] a phylum containing many bacteria typically found in microbiota surveys of the oral cavity of cats[16, 18, 22–24] and other species.[25–27]

Microbial communities inhabiting the body are shaped through a variety of intrinsic and extrinsic factors, two of which are the host’s genotype and environment. [28] Human microbiome research has indicated that genetic variation can affect the microbiota, through comparing monozygotic and dizygotic twins[29, 30] or by associating microbiota factors with specific genetic diseases.[31–33] Studies have found evidence for genotype affecting the diversity of the microbial communities found, with respect to the number of species present, as well as the taxonomic composition of the communities.[29–31, 34–36] There are even taxa that have been associated with genetic diseases, such as increased abundances of *Clostridium difficile*[31] and *Enterobacteriaceae*[32] in patients with NOD2 genotypes associated with increased risk of inflammatory bowel disease.

Research on the microbiota of humans[37, 38] and animals[39, 40] has revealed that the environment can also shape microbial communities. This has been described in multiple studies assessing the cutaneous microbiota of humans living in different environments; individuals living in more urbanized habitats tend to have a microbiota with decreased diversity,[37, 41, 42] which has been associated with an increased risk of developing allergies.[37, 43] Additionally, the presence of animals in a home has been demonstrated to alter the home microbiota,[44] so it is not surprising that direct contact with animals, including cohabitation with[22, 38, 45] or working with animals,[46–48] can also have a considerable effect on the diversity and composition on the human skin microbiota.

Evaluation of the cutaneous microbiota in various animal species, including cats, is still in its infancy, and many factors influencing the skin microbiome in animals are still unknown and should be further researched. With respect to cats, several breeds are at higher risk for certain cutaneous infectious diseases, such as Persian cats with dermatophytosis[49–51] or Devon Rex cats with *Malassezia* dermatitis;[52, 53] perhaps some of this increased risk could be related to the microbiota. With the known effect of the environment on the human skin microbiota, including a potential role in the development of allergies, the effect of environment on the feline skin microbiota should be elucidated.

Therefore, the objectives of this study were to evaluate how genotype and environment can influence the bacterial and fungal microbiota of feline skin. With the grooming habits of cats likely playing a role in microbial community composition of the skin, the oral cavity is also of interest and thus was sampled. In order to assess the effect of genotype, purebred cats of five different breeds were sampled. These cats are selectively bred to have a specific hair phenotype.[54–56] The different hair phenotypes seen may provide an altered habitat in terms of other features (e.g. lipid content, hydration, etc.), which could affect the composition and diversity of the microbiota. With respect to environment, we characterized the microbiotas of mixed genetic background cats kept strictly indoors or strictly outdoors. We hypothesized that different feline breeds would vary in their microbial communities due to the differences in genotype, resulting in phenotypic characteristics affecting the development and maintenance of the microbiota. Furthermore, we hypothesized outdoor cats would have more diverse microbial communities of a different composition relative to indoor cats, due to their exposure to a greater diversity of microbes and less stable environmental conditions.

## Materials and methods

This study was approved by the Texas A&M University (TAMU) Institutional Animal Care and Use Committee and in accordance with the relevant guidelines. Informed consent was obtained for all cats enrolled in the study.

### Sample collection

Sixty-nine cats were enrolled in this study: 11 Bengals, 10 Cornish Rexes, 4 Devon Rexes, 6 Siberians, 13 Sphynxes, 13 indoor Domestic short/medium/long hairs, and 12 outdoor Domestic shorthairs (S1 Table). Samples were taken from the axilla, dorsum, ear canal, nostril and oral cavity by rubbing each side of two Isohelix buccal swabs (Cell Projects Ltd., Kent, UK) 10 times. Both swabs were placed in a MO BIO PowerBead tube (MoBio Laboratories, Carlsbad, CA). DNA was extracted using a modified protocol with the MoBio PowerSoil DNA Extraction Kit and stored at -80°C until used. Extracted DNA from the samples and from controls (swab only and reagent only) was sent to MR DNA Lab in Shallowater, TX for sequencing on an Illumina MiSeq (Illumina, San Diego, CA). The V4 region of the 16S rRNA gene was sequenced using primers 515F: GTGYCAGCMGCCGCGGTAA and 806R: GGACTACNVGGGTWTCTAAT. The internal transcribed spacer 1 (ITS-1) region between the 18S and 5.8S rRNA genes was sequenced using primers ITS1-F: CTTGGTCATTTAGAGGAAGTAA and ITS2: GCTGCGTTCTTCATCGATGC. The sequences analyzed are available in the NCBI sequence read archive under BioProject ID PRJNA473778.

### Sequence processing

The resulting sequences were processed using QIIME 1.9.[57] Sequences were demultiplexed and open-reference OTU picking was performed with uclust.[58] For the 16s sequences, the Greengenes database (13_8 release)[59] was used with a 97% threshold of identity, and for the ITS sequences, the Findley database[10] was used. Taxa presumed to be contaminants were removed as previously described.[22]

Prior to diversity analyses, samples were rarefied to 21000 bacterial and 3800 fungal sequences in order to account for unequal sequencing depth. To evaluate alpha diversity, the Chao1, Observed OTUs, and Shannon metrics were used. Good’s coverage was used to assess sampling depth. For beta diversity the Bray Curtis, weighted UniFrac, and unweighted UniFrac metrics were used for the 16s sequences and the Bray Curtis, Abundance Jaccard and Pearson correlation metrics were used for the ITS sequences.[60]

#### Species-level classification of *Malassezia* sequences

To obtain species-level assignments for the *Malassezia* spp. sequences, the raw fungal sequences were processed using mothur[61] where they were classified with the k-nearest Neighbor algorithm and blasted against the Findley database. *Malassezia* spp. sequences were then extracted and aligned to a reference alignment of *Malassezia* spp. sequences. Species level assignments were determined using pplacer[62] and a *Malassezia* reference package.[10]

#### Quantitative PCR (qPCR)

The extracted DNA was also used for qPCRs targeting *Malassezia* spp. and *Propionibacterium* spp. For the *Malassezia* spp. qPCR, primers ITSANA-F (CGAAACGCGATAGGTAATGTG) and ITSANA-R (CAAATGACGTATCATGCCATGC)[63] were used with reactions containing 5 uL of iTaq Univeral SYBR Green Supermix (Bio-Rad Laboratories, Hercules, California), 2 uL Invitrogen UltraPure water (Invitrogen, Carlsbad, CA), 0.5 uL of each primer (Integrated DNA Technologies, Coralville, IA), and 2 uL of sample. After 3 minutes at 95°C, 39 cycles of 30 seconds at 95°C and 30 seconds at 60°C were performed, followed by a melt curve from 65°C to 95°C.

For the *Propionibacterium* spp. qPCR 20 uL reactions consisted of 10 uL iTaq Universal Probes Supermix, 5 uL Invitrogren UltraPure water, 1 uL each of oligos EUB519F (CAGCAGCCGCGGTRATA), U785R (GGACTACCVGGGTATCTAAKCC), and Prop_P ([FAM]CTTTCGATACGGGTTGACTT[BHQ-1]) (Sigma-Aldrich, St. Louis, MO) using the thermocycler conditions previously published.[64]

PCRs were run on a Bio-Rad CFX Connect Real-Time PCR Detection System, results were analyzed using Bio-Rad CFX Manager and data were normalized based on DNA concentration, as determined using the Qubit high sensitivity dsDNA assay (Qubit, London, UK).

### Statistical analysis

Statistical significance of alpha diversity results was analyzed using the Kruskal-Wallis test for overall significance and the Wilcoxon test for pairwise tests in JMP Pro 12 (SAS Institute, Cary, NC). For beta diversity results, ANOSIM on the resulting distance matrices in PRIMER 6 (PRIMER-E, Albany, New Zealand) or using the vegan package in R was used. Kruskal-Wallis tests, followed by Wilcoxon pairwise tests and Benjamini-Hochberg procedure for p-value correction[65] where appropriate, and LEfSe (with a p<0.01 considered significant) were used to analyze differential taxa abundance. For Kruskal-Wallis tests on relative taxa abundances, only taxa present at greater than 1% in at least 10 samples were tested.

## Results

To evaluate differences in the microbiota between different cat breeds, samples from Bengal, Cornish Rex, Devon Rex, Siberian, Sphynx, and indoor domestic (mixed genotype) cats were analyzed. The environment analyses included samples from indoor and outdoor domestic cats. The average Good’s coverage estimate for bacterial sequences was 0.971 and for fungal sequences 0.986.

### Bacteria

**Fig 1:**
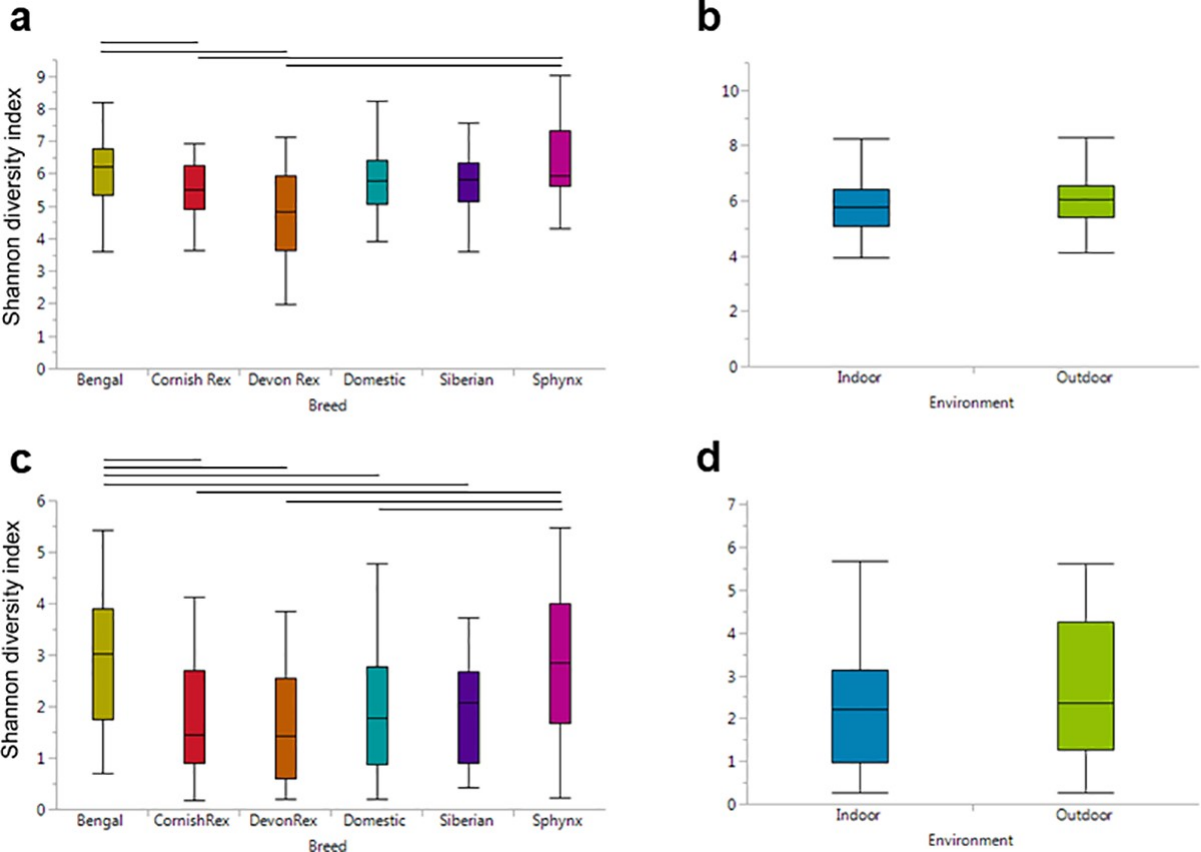
Comparing alpha diversity as measured by the Shannon diversity index between cat breeds and indoor and outdoor cats. Comparing alpha diversity of (a) bacterial communities between cat breeds (p<0.0001), (b) bacterial communities between indoor and outdoor cats (p = 0.2509), (c) fungal communities between cat breeds (p<0.0001), and (d) fungal communities between indoor and outdoor cats (p = 0.8340) using the Shannon diversity metric. Lines show significant pairwise tests where p<0.01. Sample sizes (bacterial sequencing, fungal sequencing): Bengal (54, 54), Cornish Rex (45, 45), Devon Rex(19, 19), Domestic/Indoor (55, 59), Siberian (29, 30), Sphynx (70, 69), and Outdoor (60, 58).

Cat breeds were significantly different in terms of alpha diversity (Chao1, observed OTUs, and Shannon diversity index p<0.0001; Fig 1A), which measures the number of different unique taxa identified and, with some metrics, how evenly abundant they are. Devon Rex cats had the lowest medians for alpha diversity, indicating relatively low diversity, and Bengal cats had the highest. With regards to body site, the most pronounced differences were observed for the dorsum and ear canal (Table 1). When only the dorsum was evaluated, Sphynx cats had the most diverse microbial populations (S1 Fig). Comparison of all cats based on hair length did not reveal significant differences. Alpha diversity was also not significantly different between indoor and outdoor cats, regardless of metric used or body site analyzed (Table 1; Fig 1B).

**Table 1:** Statistical analysis of alpha diversity results. P-values from Kruskal-Wallis test comparing alpha diversity results across body sites with respect to breed and environment for bacterial and fungal microbiota. P<0.05 are bolded.

	Bacteria			Fungi		
	Chao1	Observed OTUs	Shannon	Chao1	Observed OTUs	Shannon
**Breed**						
All	**<0.0001**	**<0.0001**	**<0.0001**	**<0.0001**	**<0.0001**	**<0.0001**
Dorsum	**0.0018**	**0.0007**	**0.0026**	**0.0006**	**0.0011**	**0.0013**
Ear canal	**0.0259**	**0.0043**	**0.0060**	**0.0274**	**0.0267**	**0.0165**
Groin	0.1205	0.0502	**0.0297**	0.0639	0.2155	0.1178
Nostril	0.3831	0.2762	0.8045	**0.0120**	**0.0010**	**0.0038**
Oral	0.4665	0.8634	0.5689	0.7634	0.5888	0.6466
**Environment**						
All	0.5269	0.2212	0.0836	0.7768	0.32908	0.2780
Dorsum	0.7290	0.6649	0.7728	0.4529	0.8625	0.6861
Ear canal	0.0479	0.0250	0.0210	0.4529	0.4189	0.2482
Groin	0.7416	0.8951	0.8951	0.6666	0.7119	0.5796
Nostril	0.4984	0.1567	0.6225	0.1872	0.0805	0.0559
Oral	0.1659	0.1123	0.9081	0.8703	0.5676	0.6831

**Fig 2:**
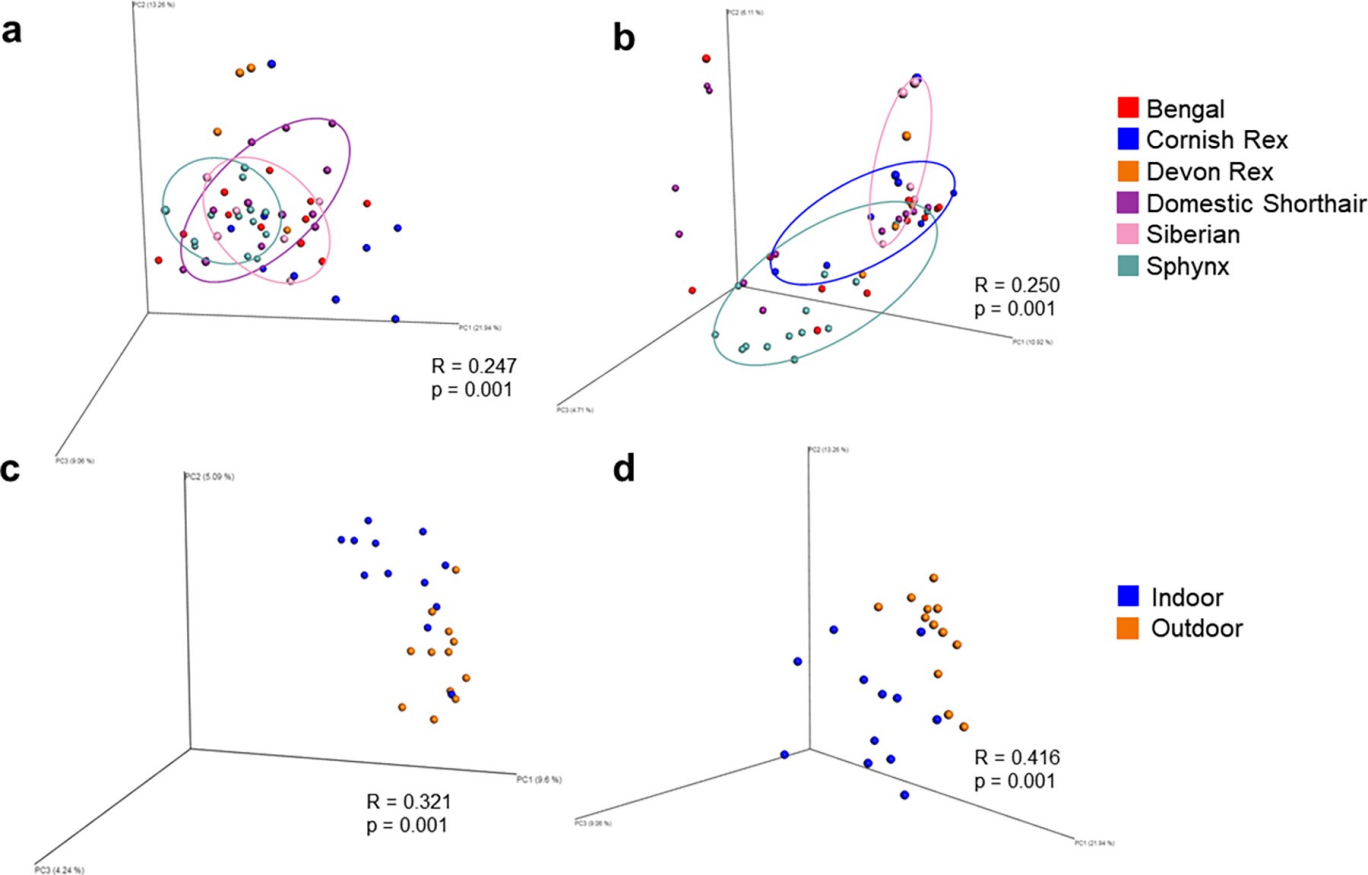
Principle coordinate analysis (PCoA) plots of beta diversity distance matrices comparing different cat breeds and indoor and outdoor cats. Comparing beta diversity of (a) bacterial communities on the dorsum between cat breeds with the weighted UniFrac metric, (b) fungal communities on the dorsum between cat breeds with the Bray-Curtis metric, (c) bacterial communities in the oral cavity between indoor and outdoor cats with the Bray-Curtis metric, and (d) bacterial communities in the oral cavity between indoor and outdoor cats with the weighted UniFrac metric. **R and p-values are from analysis of similarities (ANOSIM) test of beta diversity distance matrices**.

Beta diversity analysis revealed significant differences in the dorsum samples between cat breeds (R = 0.247 and p = 0.001, Fig 2A). The Bray-Curtis and weighted UniFrac metrics were significant, while the unweighted UniFrac was not, indicating the dissimilarity in communities is attributed to differential abundance, regardless of phylogenetic relationships between taxa. Significant differences in beta diversity between indoor and outdoor cats were only seen in the oral cavity (Table 2), with the Bray-Curtis (R = 0.321 and p = 0.001, Fig 2C) and weighted UniFrac (R = 0.416 and p = 0.001, Fig 2D) metrics.

**Table 2:** Results from ANOSIM tests on bacterial beta diversity results. Results from ANOSIM on distance matrices comparing structure of bacterial communities. R value, p-value. Results with R>0.150 and P = 0.001 are bolded.

	Bray-Curtis	Weighted UniFrac	Unweighted UniFrac
**Breed**			
All	0.099873, 0.001	0.077965, 0.001	0.046498, 0.002
** Dorsum**	**0.204435, 0.001**	**0.247425, 0.001**	0.16356, 0.001
Ear Canal	0.086077, 0.028	0.144358, 0.003	0.023431, 0.287
Groin	0.168733, 0.001	0.126788, 0.004	0.044658, 0.166
Nostril	0.12199, 0.005	0.093338, 0.023	0.040002, 0.179
Oral	0.11469, 0.011	0.11641, 0.011	0.060964, 0.089
**Environment**			
All	0.03111, 0.012	0.032157, 0.023	0.019495, 0.057
Dorsum	-0.06634, 0.981	-0.01042, 0.458	-0.07344, 0.970
Ear Canal	0.082282, 0.100	0.031982, 0.281	0.08228, 0.125
Groin	0.014865, 0.369	-0.00586, 0.466	-0.02462, 0.603
Nostril	0.05303, 0.154	0.079264, 0.088	-0.01415, 0.571
Oral	**0.32097, 0.001**	**0.416351, 0.001**	**0.19939, 0.004**

**Fig 3:**
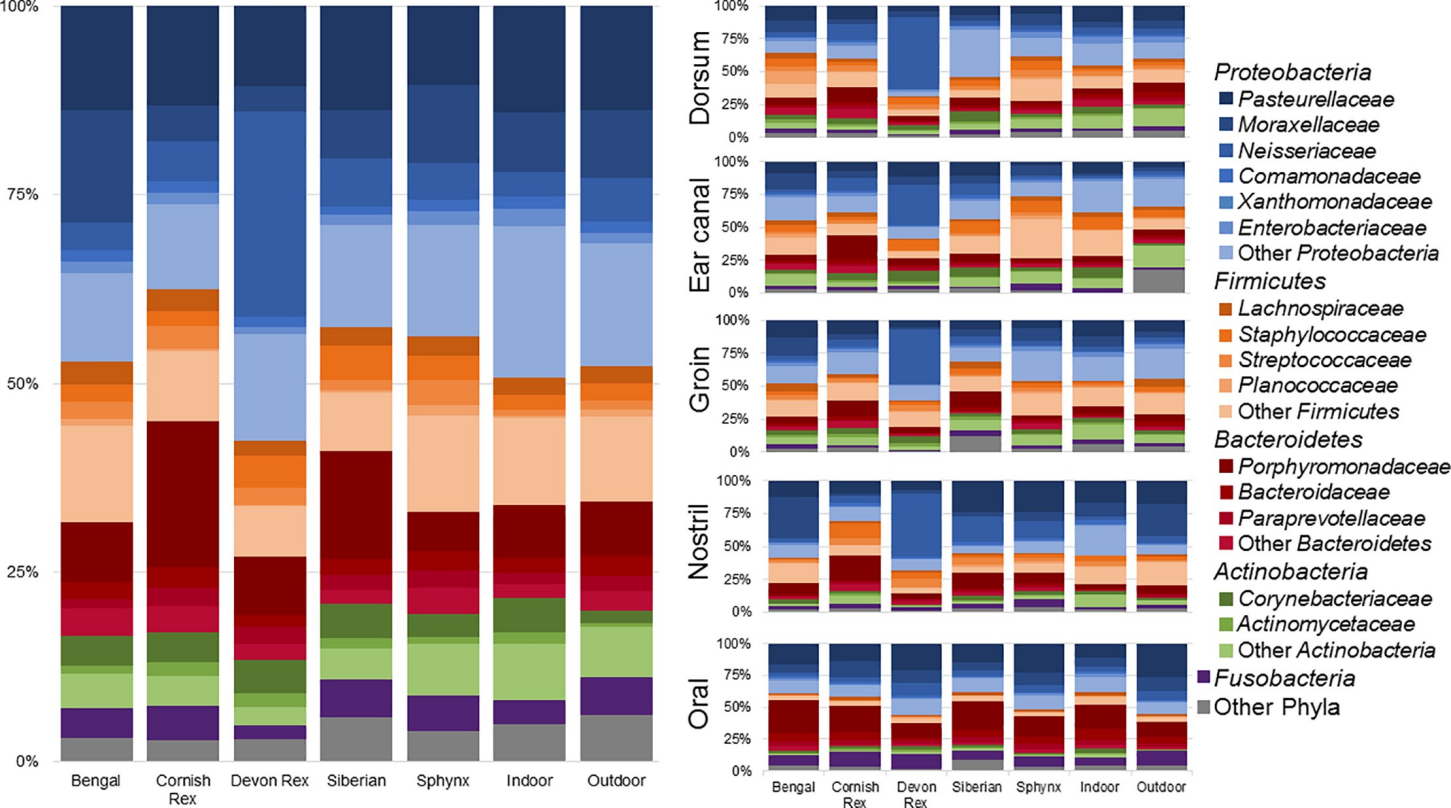
Average relative abundance of bacterial taxa. The average relative abundance of bacterial taxa across the different cat breeds and outdoor cats including all body sites and separated by body site.

The average relative abundance of bacterial taxa by sample type is shown in Fig 3. The main phyla identified were Proteobacteria (mean relative abundance = 44.03%), Firmicutes (21.04%), Bacteroidetes (16.65%), and Actinobacteria (10.38%). Some of the most abundant taxa included bacteria within the family *Pasteurellaceae* (11.14%) and from the genera *Porphyromonas* (7.40%) and *Staphylococcus* (4.79%). *Veillonellaceae*, a family of bacteria typically found in the gastrointestinal microbiota of humans and animals,[66, 67] and in lesser abundances in the human[68] and animal[1, 6] skin microbiota, was found to have significantly different relative abundances between cat breeds (Kruskal-Wallis p = 0.0004) when considering all body sites, with greater relative abundances in the ear canal of Sphynx cats (LEfSe LDA score>4.0). Additionally, other taxa such as *Porphyromonas* spp. (p = 0.0003) and *Lactobacillus s*pp. (p<0.0001) were differentially abundant across cat breeds (S3 Table).

**Fig 4:**
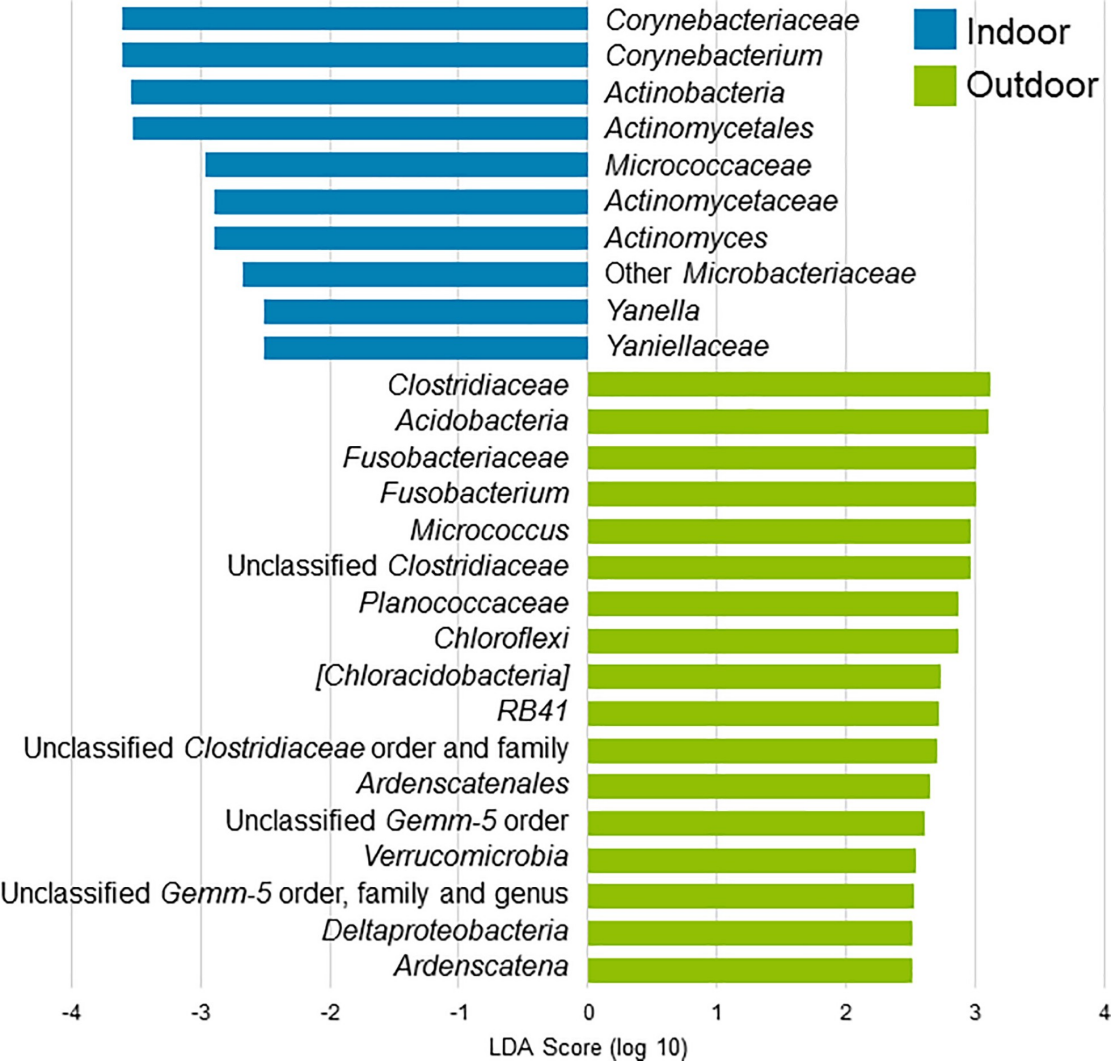
Bacteria found to be differentially abundant between indoor and outdoor cats as determined by LEfSe. When comparing all body sites, many taxa were identified as differentially abundant between indoor and outdoor cats.

Many bacterial taxa were also found to be differentially abundant between indoor and outdoor cats when considering all body sites (Fig 4 and S2A Fig, without oral cavity), and when considering only the nostril samples (S2C Fig). One bacterial genus identified to be differentially abundant when considering all body sites was *Corynebacterium* spp.; greater relative abundance was identified in samples from indoor cats (average relative abundance of 5.7% in indoor cats and 1.9% in outdoor; LEfSe |LDA| > 3.5; Wilcoxon test p = 0.0043).

Because of the relevance of *Propionibacterium* spp. in the cutaneous microbiota of humans and the known inability of the sequencing primers used in this study to target this genus accurately,[69, 70] a qPCR for the genus was performed to investigate its abundance on feline skin. No significant differences in *Propionibacterium* spp. abundance between the different cat breeds or between indoor and outdoor cats were found (S3A Fig).

### Fungi

As was found with the bacterial microbiota, alpha diversity of fungal communities was significantly different between different cat breeds when considering all body sites, with Sphynx and Bengal cats having the highest diversity (p<0.0001, across all 3 metrics) (Fig 1C). Furthermore, with regards to body site, cat breeds were significantly different on the dorsum, ear canal and nostril (Table 1). Similar to the bacterial data, no significant difference in alpha diversity was found in the fungal sequences between indoor and outdoor cats (Table 1; Fig 1D). When hair length was analyzed, significant differences were observed when evaluated all body sites today and when analyzing only the dorsum with all alpha diversity metrics (all p<0.01). Breeds with short (DSH) and very short (Cornish Rex, Devon Rex, and Sphynx cats) seemed to have more diverse communities relative to cats with long or medium hair (S7 Fig).

Both the dorsum (Bray-Curtis R = 0.250, p = 0.001; Pearson R = 0.221 and p = 0.001; Table 3 and Fig 2B) and groin (Bray-Curtis R = 0.244, p = 0.001) were body sites where significant differences in beta diversity were found between cat breeds. Regardless of metric used or body sites analyzed, no significant clustering was found between indoor and outdoor cat samples (Table 3).

**Table 3:** Results from ANOSIM tests on fungal beta diversity results. Results from ANOSIM on distance matrices comparing structure of fungal communities. R value, p-value.

	Bray-Curtis	Pearson	Jaccard
**Breed**			
All	0152456, 0.001	0.125037, 0.001	0.074513, 0.001
** Dorsum**	**0.250435, 0.001**	**0.221035, 0.001**	0.03777, 0.198
Ear Canal	0.178865, 0.001	0.135659, 0.001	0.067723, 0.059
** Groin**	**0.243501, 0.001**	0.170137, 0.001	0.092201, 0.022
Nostril	0.039623, 0.197	0.005078, 0.457	-0.03487, 0.77
Oral	-0.04238, 0.834	-0.01625, 0.662	-0.02459, 0.707
**Environment**			
All	0.011423, 0.079	0.014186, 0.028	-0.00166, 0.513
Dorsum	-0.04267, 0.889	-0.04682, 0.947	-0.03809, 0.795
Ear Canal	0.015152, 0.254	0.017677, 0.21	-0.03388, 0.725
Groin	0.014525, 0.312	0.018908, 0.285	0.022414, 0.322
Nostril	-0.056765, 0.946	-0.01471, 0.591	0.003754, 0.454
ral	0.016049, 0.333	0.051852, 0.111	-0.01289, 0.537

**Fig 5:**
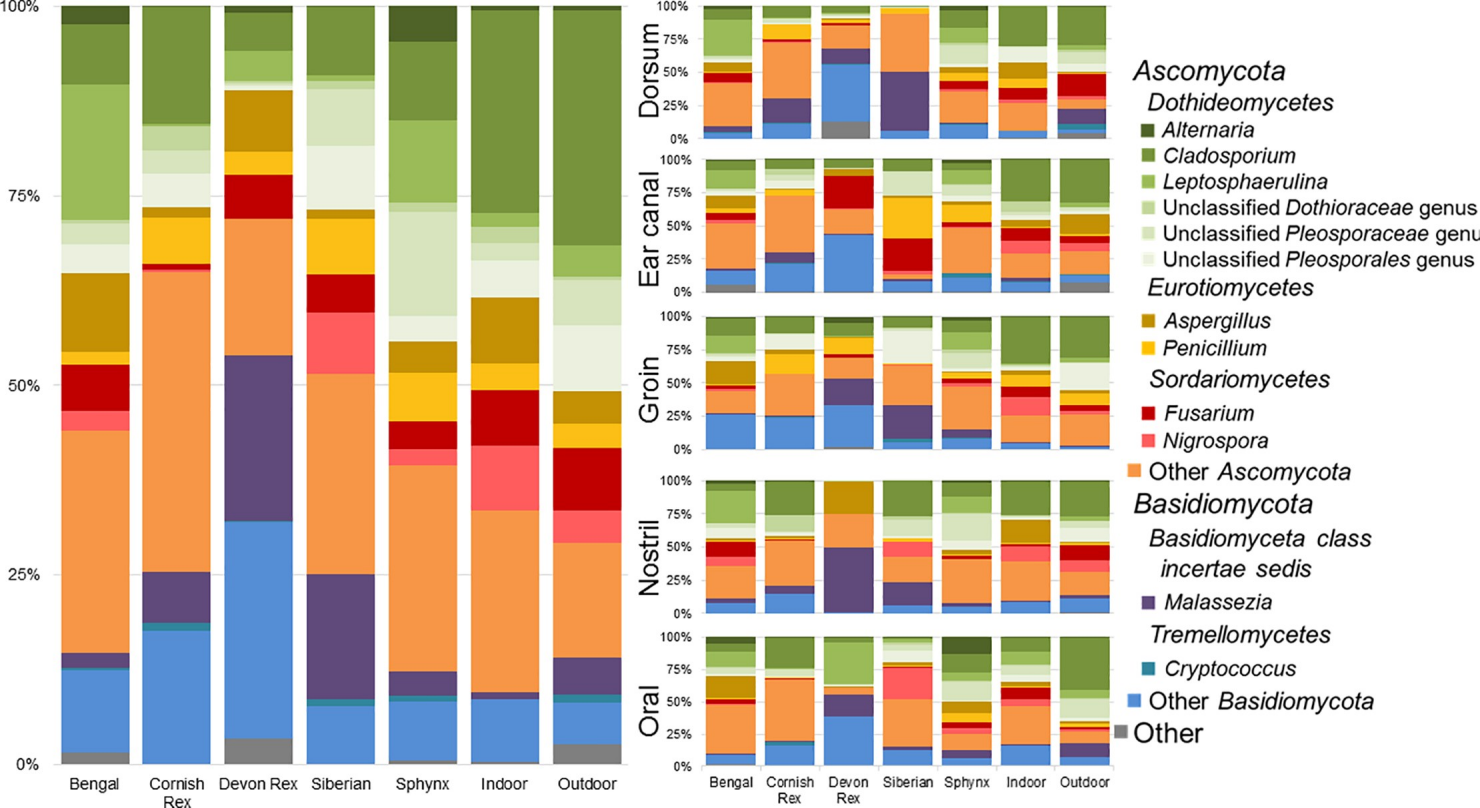
Average relative abundance of fungal taxa. The average relative abundance of fungal taxa across the different cat breeds and outdoor cats including all body sites and separated by body site.

Fig 5 shows a summary of the most abundant fungal taxa. Relative to the bacterial microbiota, the composition of the fungal communities was more variable between cat breeds. Some of the most abundant genera included *Cladosporium* spp. and *Malassezia* spp. When comparing all samples across cat breeds many taxa were differentially abundant (S4 Table), including *Alternaria* spp. (Kruskal-Wallis p = 0.0064), *Aspergillus* spp. (p = 0.0026), and *Malassezia* spp. (p = 0.0026). Looking at differences in taxa abundance at the body site level, the dorsum had the most significant changes at different taxonomic levels, followed by the nostril and groin. This was particularly evident in the relative abundance of *Malassezia* spp. (p = 0.0096) and *Alternaria* spp. (p = 0.0078) in samples collected from the dorsum.

In comparing indoor and outdoor cats, taxa that were differentially abundant were found when including all sites except the oral cavity (S2B Fig), and when considering the dorsum (S2D Fig) and the nostril samples (S2E Fig) separately. Ustilaginomycetes and Ustilaginales,[71] taxa containing primarily plant pathogens, were found to be increased on outdoor cats in the analyses including all body sites except oral cavity, and in samples from the dorsum (<0.0001% in indoor cats, 1.09% in outdoor cats). Two of three phyla were identified to be differentially abundant in the nostril samples: Ascomycota (Wilcoxon test p = 0.0303, higher in indoor) and Basidiomycota (p = 0.01934, higher in outdoor), in addition to sequences that were unassigned to any phylum (p = 0.0224).

### Malassezia sequence analysis

Due to the significant findings in regards to *Malassezia* spp. abundance and previous research into their differential abundance between cat breeds[53], species-level classification of these sequences was performed. *M*. *restricta* and *M*. *globosa* were the most prevalent species with average relative abundances (relative to total *Malassezia* spp. sequences) of 37.0% and 23.9%, respectively, across all samples. Sequences also matched *M*. *slooffiae*, *M*. *furfur*, *M*. *nana*, *M*. *pachydermatis*, *M*. *dermatis*, *M*. *sympodialis*, *M*. *japonica*, *M*. *obtusa*, *and M*. *yamatoensis*, with average relative abundances ≤8.6% (S5 Table), and with an average of 17.0% of the *Malassezia* sequences not classified to the species level. Although *M*. *slooffiae* accounted for 25.4% of the total *Malassezia* spp. sequences, five samples had the majority of these sequences (sequence range: 13260 to 103995), which were from various body sites from two non-cohabiting Cornish Rex cats (6 and 16). As shown in S4 Fig, *Malassezia* spp. abundance is significantly different between cat breeds (p = 0.0026), with Devon Rex cats having the highest abundance. No significant difference in abundance of any specific *Malassezia* species were found between the different cat breeds or when comparing indoor and outdoor cats.

Quantitative PCR targeting *Malassezia* spp. revealed significant differences in abundance between the different cat breeds (p<0.0001), but not the indoor and outdoor cats (S3B Fig). *Malassezia* spp. were significantly more abundant in the domestic shorthair cats relative to all other feline breeds (p≤0.0016).

### Other factors affecting the microbiota

Samples from the domestic (mixed genotype) indoor cats were assessed for influence of age group and sex. Females were found to have more diverse fungal communities in the oral cavity (Wilcoxon on Chao1 p = 0.0201 and Shannon diversity index p = 0.0201) and on the skin (Chao1 p = 0.0153, observed OTUs p = 0.0443). Additionally, senior cats (7+ years) had more diverse bacterial (observed OTUs p = 0.0327) and fungal (Chao1 p = 0.0416) communities on the skin compared to adult cats (1–7 years). Only the oral cavity was affected by either of these factors in terms of beta diversity, with bacterial communities being influenced by age group (ANOSIM on Bray-Curtis dissimilarity R = 0.2332, p = 0.037) and the fungal communities by sex (Pearson correlation R = -0.2602, p = 0.986). Some taxa were found to be differentially abundant on the skin with respect to these factors (LEfSe, p<0.01), however many had relatively low LDA scores indicating minor impacts on differences between the groups and/or are not of known importance in the oral cavity or skin microbiota (S6 Table).

## Discussion

This is the first study evaluating the effect of breed and environment on the feline skin and oral microbiota. Many of the cat breeds that are recognized today were developed through selection of specific hair coats. Mutations that contribute to the different hair coats have been identified and can result in a reduced coat, almost to the point of being considered”hairless”, such as in the Sphynx, or short wavy hair, such as in the Cornish Rex.[54–56] The difference in hair coats, and perhaps variation in other features of the skin (lipid production, water content), may be responsible for altered microhabitats that could support different microbial communities. For example, Devon Rex cats are thought to develop seborrheic dermatitis involving the lipophilic yeast *Malassezia* spp. more often than other breeds.[52, 53] Currently this has not been further investigated but perhaps genetic mutations that affect lipid content or epidermal maturation in Devon Rex cats could explain this. In the results presented, the different cat breeds sampled showed differences with respect to the diversity of their bacterial and fungal communities (Fig 1) and showed that individual cats did cluster with others of their same breed (Fig 2).

One aspect of the cat breeds we thought would contribute to the microbiome was the length of the hair coat. However, when cats were grouped based on this factor (Cornish Rex, Devon Rex, and Sphynx = “very short”, Bengals and DSH =“short”, DMH =“medium”, Siberian and DLH =“long”) significant differences were only found in evaluating fungal alpha diversity (S7 Fig) and in terms of some differentially abundant taxa in specific body sites (S7 Table). Considering we observed many other differences between cat breeds, there are likely other physiologic differences, likely influenced by genetics, which play a role. If hair length were the only influencing difference in physiology that contributes to the microbiome, we would expect the short-haired Cornish Rex, Devon Rex cats, and almost hairless Sphynx cats to harbor a different microbiome from long haired Siberian cats. In the results presented however, this is not observed; comparing alpha diversity showed that Sphynx cats had higher Shannon diversity than all three of these other breeds, with significantly more diverse communities compared to the Cornish and Devon Rex cats, but not the Siberian cats (Fig 1A and 1C). To our knowledge, no studies have evaluated the cutaneous microhabitat in terms of pH, hydration, lipid composition, etc. of cats or comparing between cat breeds that would allow for a clear understanding of which features may be responsible for the microbiome differences observed. Once these data are available, it would be possible to re-analyze the data in the present study, in the context of these physiological differences.

The environment also has a role in shaping the microbiota. In terms of composition, many taxa were found to be differentially abundant. For example, outdoor cats harbored higher relative abundances of two fungal plant pathogen taxa, Ustilaginomycetes and Ustilaginales (S2B and S2D Fig). Bacterial taxa were also found to have significant differences in relative abundance (S2A and S2E Fig), however many of those were present in relatively low abundances, so their impact is not clear at this point. One bacterial taxon with differential abundance was *Corynebacterium* spp.; this microbe is found in relatively high abundances on human skin,[72] so its higher abundance on indoor cats could be due to their closer contact with human microbiota. Interestingly, environment also affected beta diversity of bacterial communities, but only in the oral cavity (Table 2). Perhaps this could be attributed partially to diet, since outdoor cats have access to a greater diversity of food sources. Contrary to what we hypothesized, outdoor cats did not have a more diverse skin microbiota than indoor cats in terms of the number of different taxa found (Fig 1B and 1D). Considering the sharing of microbiota that we know to exist between cohabiting humans and animals[22, 45, 48] and humans and the environment,[37, 38] maybe larger differences between indoor and outdoor cats were not seen because indoor-only cats also come into regular contact with environment-associated microbes through the microbial communities that are carried in the air or on their owners. The grooming habits of cats also likely contributes to these findings; perhaps the oral cavity acts as a collection site, collecting the microbes from the environment that are only transiently associated with the skin. Alternatively, maybe the microbiota exchange occurs in the opposite direction, with the oral cavity microbes being transplanted to the skin and potentially stabilizing the cutaneous communities.

While environment, and to a lesser degree breed, had an effect on the oral bacterial microbiota, there were no significant differences in the oral mycobiota between either indoor and outdoor cats or the different cat breeds. As mentioned above, diet likely also has a role in influencing the oral cavity microbiome. Indoor cats are most often fed a commercial diet, whereas outdoor cats may receive a commercial diet, but also have access to small mammals, insects, plants, etc. Another study has found diet can affect the feline oral microbiome; cats fed a dry food diet had a more diverse oral communities relative to cats fed a wet food diet, which could be attributed to the higher relative abundance of several taxa.[24] Within our study, we were not able to obtain information regarding diet for all cats, especially outdoor cats, preventing us from analyzing the influence of diet.

One particularly interesting finding across the mycobiota of different cat breeds was the relative abundance of *Malassezia* spp. In our NGS data, we had similar results to those of Bond et al., with Devon Rex cats having the highest abundance of *Malassezia* spp. (p = 0.0003) compared to the other cat breeds sampled (S4 Fig).[53] In our qPCR data, while *Malassezia* spp. were not significantly more abundant in the Devon Rex cats compared to the other breeds, these cats did have the highest median abundance (S3B Fig). This lack of agreement may be due to amplification bias, meaning the two primer pairs do not equally amplify all species.[73] In addition to further investigating differential *Malassezia* spp. abundance across cat breeds, we were also interested in describing the abundance of different *Malassezia* species. Previously, *M*. *pachydermatis* was found to be the most abundant on feline skin,[53] however in the presented NGS data, *M*. *restricta* and *M*. *globosa* were the most abundant species across all cat breeds and both indoor and outdoor cats (S5 Table). The previous study utilized a culture-based technique to describe the *Malassezia* populations on feline skin which likely contributes to the different findings, due to the fastidious nature of some *Malassezia* species.[74] These findings further support differential *Malassezia* spp. abundance across breeds and warrant further research into this yeast’s role on feline skin.

With next generation sequencing studies, the bias introduced by primer pair choice should be considered and primer sets that best capture the microbiota of interest should be used when possible.[70, 75] With the bacterial primer set used in the presented study, *Propionibacterium* spp. abundance is not accurately represented.[69] However, previous studies have indicated *Propionibacterium* spp. may not be as prominent in the skin microbiota of cats and dogs,[1, 4] so the lack of *Propionibacterium* spp. sequences may not be as impactful as in human studies. Perhaps there is a lack of *Propionibacterium* spp. on canine and feline skin, which could be attributed to physiological differences of their skin relative to human skin, however more research describing the normal microhabitat of companion animal skin are needed to provide better support for this.[76, 77] In order to describe the *Propionibacterium* spp. populations on feline skin, we used a quantitative PCR, but did not find differences between the cat breeds or indoor and outdoor cats (S3A Fig). Although studies have focused on optimizing primers for human skin studies, this has not yet been done for cats or dogs. Future sequencing projects utilizing other primers sets and larger cohorts would add to the existing characterization of the feline cutaneous microbiota. Additionally, since we know their communities and skin habitats are different from humans, studies identifying optimal sequencing primers for animal skin microbiota surveys should be performed.

Additional studies looking at other breeds as well as other influencing factors should be performed to better understand the importance of the findings presented. In this study, analysis of only the indoor domestic cats indicated some significant differences with respect to age and sex, however the sample numbers used to perform these comparisons were low and no differences of seemingly biological significance were observed; further studies focused on these factors, while controlling for others, would be more informative on their impact. In addition to considering what differences may exist, we also need to understand why these differences exist and the impact of their effects. For example, perhaps some of the differences with environment are only transient and simply due to exposure to a more varied microbial habitat outdoors; longitudinal studies would help discern this. Additionally, this study included cats from a relatively small area; surveys encompassing other geographic ranges of different climates and types of outdoor environments would add to our knowledge of the environment’s influence on the feline skin microbiota. Lastly, studies sequencing the host genome along with the skin microbiota, would allow for clearer associations between the feline genotype and the microbial communities inhabiting their skin.

## Conclusion

Our findings demonstrate that the breed and, to a lesser degree, the environment, play a significant role in shaping the feline cutaneous microbiota. The many differences in the microbiota of different cat breeds are likely due to innate features of the different cat breeds, such as hair coat, that may support growth of different microbial communities. Grooming is likely an important influence on the feline skin microbiota, and may overshadow other factors known to be relevant for humans and other animals; research into how grooming shapes the microbiota may allow us to better understand the importance of other factors.

## Supporting information

S1 Fig Significant differences in Shannon diversity index between cat breeds by site. Differences were found in the Shannon diversity index when comparing the (a) bacterial sequences in the dorsum, ear canal, and groin and when comparing the (b) fungal sequences in the dorsum, ear canal, and nostril.
(TIF)

S2 Fig Taxa found to be differentially abundant between indoor and outdoor cats as determined by LEfSe. When comparing all body sites but the oral cavity, many (a) bacteria and (b) fungi were identified as differentially abundant between indoor and outdoor cats. Additionally, differentially abundant taxa were found when looking at just the bacterial sequences in the (c) nostril samples and the fungal communities in the (d) dorsum and (e) the nostril.
(TIF)

S3 Fig Results of *Propionibacterium* spp. and *Malassezia* spp. qPCRs. (a) With the *Propionibacterium* spp. qPCR, no significant differences were found between cat breeds (P = 0.5965) or between indoor and outdoor cats (p = 0.3808). (b) Significant differences in *Malassezia* spp. as quantified by qPCR were found between the different cat breeds (p<0.0001) but not between indoor and outdoor cats (p = 0.5803). Plots do not show points for extreme outliers, however statistical analyses and box plots were made when including the outliers. Lines show significant pairwise tests where p<0.01.
(TIF)

S4 Fig Average relative abundance of *Malassezia* spp. on feline skin. The height of the bar shows the average relative abundance of *Malassezia* spp. in each sample type, while the specific species are shown in terms of median relative abundance. *M*. *restricta* and *M*. *globosa* were the most abundant. Lines show significant pairwise tests of *Malassezia* spp. abundance where p<0.05.
(TIF)

S5 Fig Relative abundance of bacterial taxa in each sample. (TIF)

S6 Fig Relative abundance of fungal taxa in each sample. (TIF)

S7 Fig Evaluating the influence of hair length on alpha diversity. Evaluating the influence of hair length on (a) bacterial alpha diversity did not reveal any differences, but significant differences were observed in two metrics of (b) fungal alpha diversity. Cats with short (DSH and Bengal cats) and very short (Cornish Rex, Devon Rex, and Sphynx cats) hair have significantly more diverse communities than long haired cats (DLH and Siberian cats) with the Chao1 and observed OTUs alpha diversity metrics. Bars indicated significant pairwise comparisons where the p<0.05.
(TIF)

S1 Table Signalment of sample cohort. (PDF)

S2 Table P-values from pairwise Kruskal-Wallis tests on alpha diversity between cat breeds. P<0.05 are bolded.
(PDF)

S3 Table Relative abundance of bacterial genera present at 1% in at least 10 samples. Average (min-max), P<0.05 are bolded.
(PDF)

S4 Table Relative abundance of fungal genera present at 1% in at least 10 samples. Average (min-max). P<0.05 are bolded.
(PDF)

S5 Table Relative abundance of Malassezia species. Average, median (min-max).
(PDF)

S6 Table Taxa determined to be differentially abundant on the skin across age groups and sex with LEfSe (LDA>2.5, p<0.01). (PDF)

S7 Table Taxa determined to be differentially abundant on the skin across hair length groups with LEfSe (LDA>2.5, p<0.01). (DOCX)
