# Supplementary figures and images for: The feline cutaneous and oral microbiota are influenced by breed and environment
Source: PLoS One. 2019 Jul 30;14(7):e0220463. doi: 10.1371/journal.pone.0220463 (PMC6667137; doi:10.1371/journal.pone.0220463)

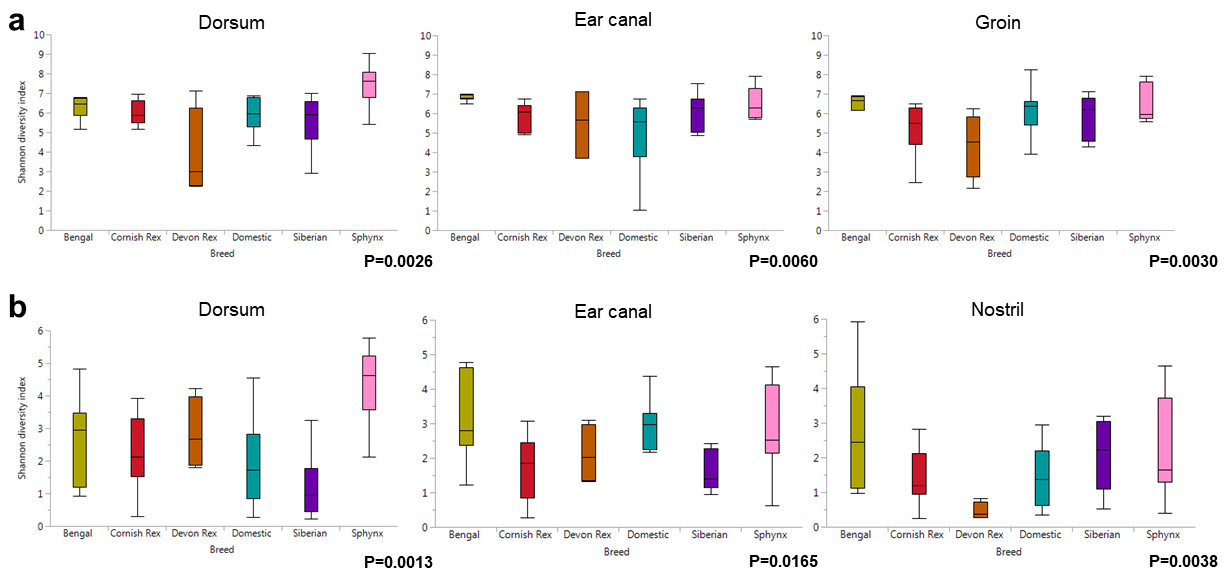

Supplement: S1 Fig — Differences were found in the Shannon diversity index when comparing the (a) bacterial sequences in the dorsum, ear canal, and groin and when comparing the (b) fungal sequences in the dorsum, ear canal, and nostril. (TIF) [file pone.0220463.s001.tif]

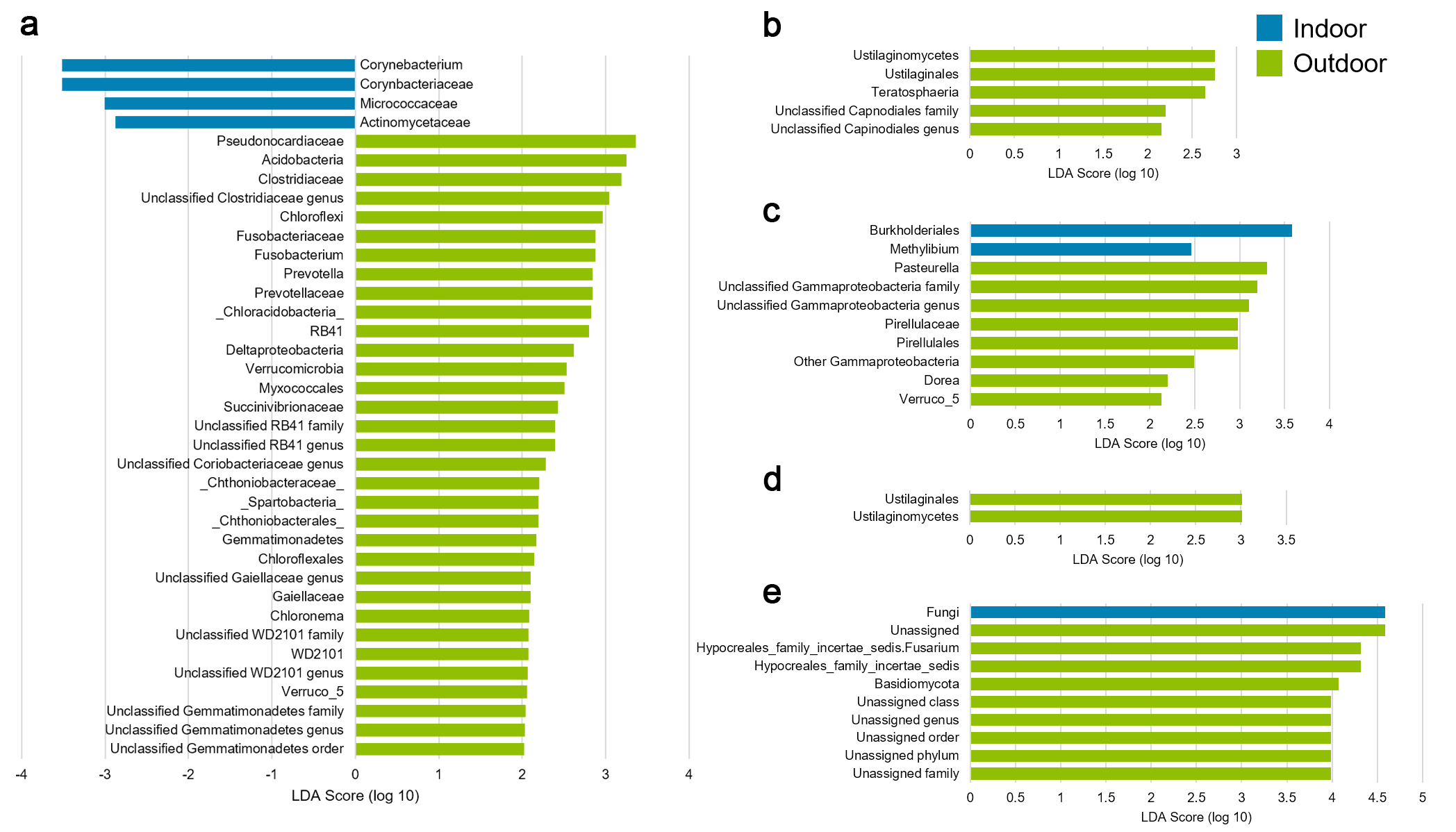

Supplement: S2 Fig — When comparing all body sites but the oral cavity, many (a) bacteria and (b) fungi were identified as differentially abundant between indoor and outdoor cats. Additionally, differentially abundant taxa were found when looking at just the bacterial sequences in the (c) nostril samples and the fungal communities in the (d) dorsum and (e) the nostril. (TIF) [file pone.0220463.s002.tif]

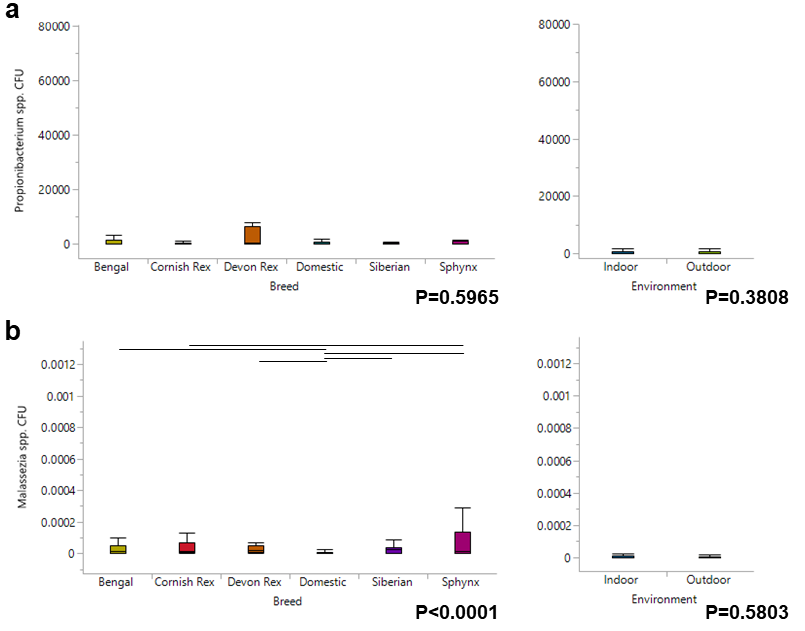

Supplement: S3 Fig — (a) With the Propionibacterium spp. qPCR, no significant differences were found between cat breeds (P = 0.5965) or between indoor and outdoor cats (p = 0.3808). (b) Significant differences in Malassezia spp. as quantified by qPCR were found between the different cat breeds (p<0.0001) but not between indoor and outdoor cats (p = 0.5803). Plots do not show points for extreme outliers, however statistical analyses and box plots were made when including the outliers. Lines show significant pairwise tests where p<0.01. (TIF) [file pone.0220463.s003.tif]

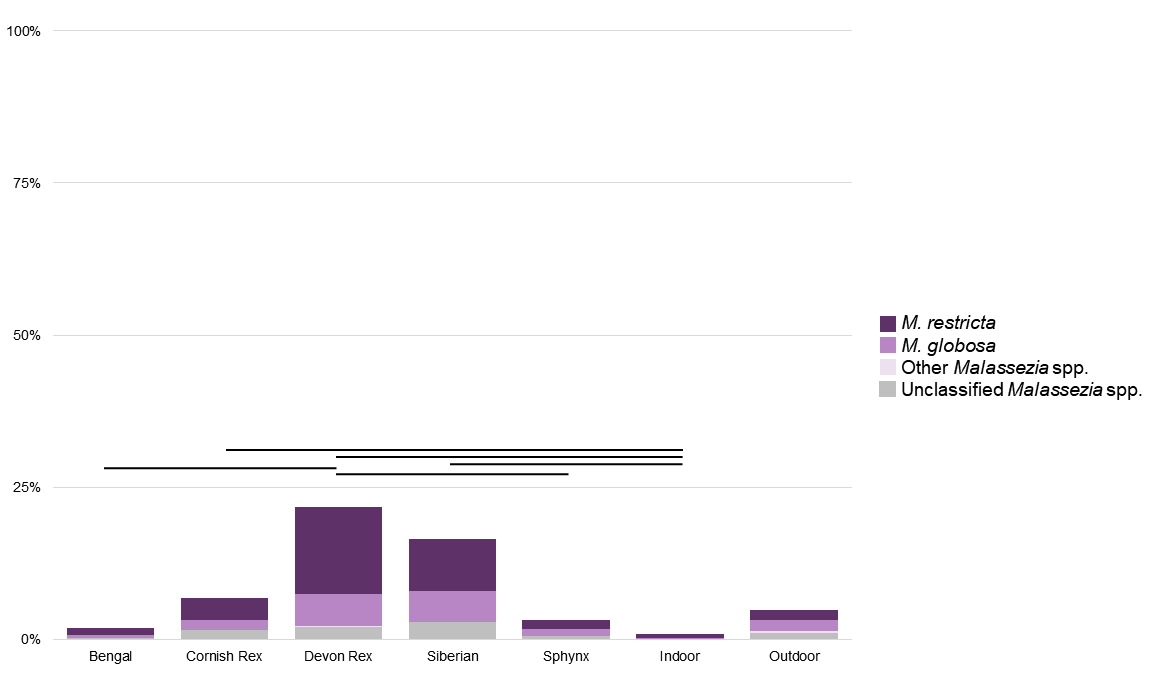

Supplement: S4 Fig — The height of the bar shows the average relative abundance of Malassezia spp. in each sample type, while the specific species are shown in terms of median relative abundance. M. restricta and M. globosa were the most abundant. Lines show significant pairwise tests of Malassezia spp. abundance where p<0.05. (TIF) [file pone.0220463.s004.tif]

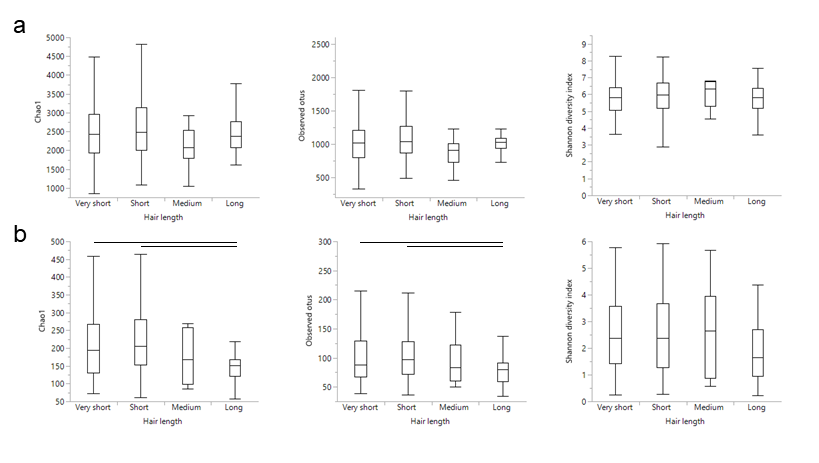

Supplement: S7 Fig — Evaluating the influence of hair length on (a) bacterial alpha diversity did not reveal any differences, but significant differences were observed in two metrics of (b) fungal alpha diversity. Cats with short (DSH and Bengal cats) and very short (Cornish Rex, Devon Rex, and Sphynx cats) hair have significantly more diverse communities than long haired cats (DLH and Siberian cats) with the Chao1 and observed OTUs alpha diversity metrics. Bars indicated significant pairwise comparisons where the p<0.05. (TIF) [file pone.0220463.s007.tif]
