# Supplementary material for: The feline cutaneous and oral microbiota are influenced by breed and environment
Source: PLoS One. 2019 Jul 30;14(7):e0220463. doi: 10.1371/journal.pone.0220463 (PMC6667137; doi:10.1371/journal.pone.0220463)
Supplement: S1 Table — (PDF) [file pone.0220463.s008.pdf]

Table S1. Signalment of sample cohort.

Cat #	Breed	Sex	Age	Indoor/outdoor (type)
1	Sphynx	CM	1y	100/0
2	Cornish Rex	M	7m	100/0
3	Siberian	SF	6y	100/0
4	Sphynx	F	5y	100/0
5	Sphynx	F	10m	100/0
6	Cornish Rex	SF	16y	100/0
7	Cornish Rex	M	4m	100/0
8	Devon Rex	M	9m	100/0
9	Cornish Rex	CM	3y	100/0
11	Bengal	SF	4y	100/0
12	Sphynx	F	2y	100/0
13	Cornish Rex	CM	10y	100/0
14	Siberian	CM	6y	99/1 (patio)
15	Siberian	SF	6y	99/1 (patio)
16	Cornish Rex	CM	15y	100/0
17	Bengal	CM	12y	100/0
18	Sphynx	SF	2.5y	100/0
19	Bengal	CM	9y	100/0
20	Bengal	SF	5y	100/0
21	Cornish Rex	CM	3y	100/0
22	Cornish Rex	CM	3y	100/0
23	Sphynx	F	3y	100/0
24	Sphynx	CM	4y	100/0
25	Sphynx	CM	4y	100/0
26	Bengal	SF	6y	30/70 (trees, grass, weeds)
27	Sphynx	CM	4y	100/0
28	Sphynx	M	2y	100/0
29	Sphynx	CM	3.5y	100/0
30	Bengal	M	3y	100/0
31	Bengal	F	3y	100/0
32	Devon Rex	SF	1y	100/0
33	Devon Rex	SF	10y	98/2 (grasses, patio)
34	Sphynx	SF	5m	100/0
36	Siberian	CM	3y	100/0
37	Sphynx	SF	5m	100/0
39	Devon Rex	SF	1.5y	100/0
40	Bengal	F	3m	100/0
41	Bengal	M	3m	100/0
43	Cornish Rex	CM	14y	100/0
45	Bengal	M	1y	100/0

51	Siberian	SF	4y	100/0
52	Siberian	SF	4y	100/0
53	Cornish Rex	SF	4y	100/0
54	Bengal	CM	5y	100/0
Indoor and outdoor cats				
Indoor 1	DLH	CM	6y	100/0
Indoor 2	DSH	F	4m	100/0
Indoor 3	DSH	M	1y 4m	100/0
Indoor 4	DSH	M	10y	100/0
Indoor 5	DSH	F	3y	100/0
Indoor 6	DMH	F	6y	95/5 (trees, grasses, weeds)
Indoor 7	DSH	CM	3y	100/0
Indoor 8	DSH	SF	9y	100/0
Indoor 9	DSH	SF	11y	98/2 (driveway)
Indoor 10	DSH	F	12y	100/0
Indoor 11	DSH	M	13y	100/0
Indoor 12	DSH	SF	3y	100/0
Indoor 13	DMH	CM	5y	100/0
Outdoor 1	DSH	SF	13y 3m	0/100
Outdoor 2	DSH	SF	13y 3m	0/100
Outdoor 4	DSH	SF	1y	0/100
Outdoor 5	DSH	F	1y	0/100
Outdoor 6	DSH	M	9.5m	0/100
Outdoor 7	DSH	M	10m	0/100
Outdoor 8	DSH	M	10m	0/100
Outdoor 9	DSH	F	2y	0/100
Outdoor 10	DSH	M	2.5y	0/100
Outdoor 11	DSH	F	2y	0/100
Outdoor 12	DSH	F	1.5y	0/100
