# Supplementary material for: The feline cutaneous and oral microbiota are influenced by breed and environment
Source: PLoS One. 2019 Jul 30;14(7):e0220463. doi: 10.1371/journal.pone.0220463 (PMC6667137; doi:10.1371/journal.pone.0220463)
Supplement: S2 Table — P<0.05 are bolded. (PDF) [file pone.0220463.s009.pdf]

Table S2. P-values from pairwise Kruskal-Wallis tests on alpha diversity between cat breeds. P<0.05 are bolded.

	Bacteria			Fungi		
**Comparison**	Chao1	Observed OTUs	Shannon	Chao1	Observed OTUs	Shannon
Bengal vs Cornish Rex	**0.001807**	**0.000952**	**0.005646**	0.064638	**0.006272**	**0.029991**
Bengal vs Devon Rex	**0.006034**	**0.002365**	**0.004035**	**0.009591**	**0.004491**	0.133539
Bengal vs Indoor	**0.001805**	**0.000804**	**0.024916**	0.326408	**0.045784**	0.113762
Bengal vs Siberian	**0.031257**	0.071091	0.092487	**0.000149**	**0.000305**	**0.027979**
Bengal vs Sphynx	0.570611	0.876334	0.943228	0.748411	0.977934	0.503447
Cornish Rex vs Devon Rex	0.578139	0.443209	0.136106	0.218611	0.477304	0.964838
Cornish Rex vs Indoor	0.835341	0.814794	0.411682	0.401696	0.332903	0.658185
Cornish Rex vs Siberian	0.392441	0.310163	0.419229	0.064769	0.446317	0.790466
Cornish Rex vs Sphynx	**0.008421**	**0.002674**	**0.006505**	**0.029194**	**0.004844**	**0.002707**
Devon Rex vs Indoor	0.609884	0.599932	0.055562	0.060383	0.149172	0.730382
Devon Rex vs Siberian	0.382405	0.232065	0.053137	0.845439	0.967255	0.770921
Devon Rex vs Sphynx	**0.027825**	**0.006384**	**0.005130**	**0.006701**	**0.003864**	**0.025404**
Indoor vs Siberian	0.292488	0.139056	0.848809	**0.004866**	**0.049773**	0.648264
Indoor vs Sphynx	**0.004992**	**0.000987**	**0.029604**	0.210413	**0.037494**	**0.012794**
Siberian vs Sphynx	0.210543	0.105923	0.093746	**0.000277**	**0.000498**	**0.002019**
