# Supplementary material for: The feline cutaneous and oral microbiota are influenced by breed and environment
Source: PLoS One. 2019 Jul 30;14(7):e0220463. doi: 10.1371/journal.pone.0220463 (PMC6667137; doi:10.1371/journal.pone.0220463)
Supplement: S3 Table — Average (min-max), P<0.05 are bolded. (PDF) [file pone.0220463.s010.pdf]

Table S3. Relative abundance of bacterial genera present at 1% in at least 10 samples. Average (min-max), P<0.05 are bolded.

Taxon					P-value		Sample type						
Phylum	Class	Order	Family	Genus	Breed	Environ-ment	Bengal	Cornish Rex	Devon Rex	Siberian	Sphynx	Indoor	Outdoor
Thermi	Deinococci	Deinococcales	Deinococcaceae	Deinococcus	0.1001	0.6544	0.2 (0-2.7)	0.1 (0-1.5)	0.1 (0-0.6)	0.2 (0-1.8)	0.2 (0-2.1)	0.8 (0-11)	0.2 (0-3.5)
Acidobacteria	Acidobacteria-6	iii1-15			0.3141	0.2504	0.3 (0-3.9)	0.2 (0-2.9)	0.1 (0-0.6)	0.7 (0-4.3)	0.1 (0-0.8)	0.2 (0-2)	0.2 (0-2)
			RB40		0.1359	0.7162	0.2 (0-2.7)	0.3 (0-2)	0.1 (0-1.5)	0.1 (0-0.6)	0.4 (0-4.2)	0.4 (0-5)	1.1 (0-8.2)
Actinobacteria	Actinobacteria	Actinomycetales	Actinomycetaceae	Actinomyces	**0.0164**	0.0655	3.7 (0.1-18.8)	3.8 (0.1-16.6)	3.4 (0.2-17.7)	3.8 (0.2-18.2)	3.7 (0.1-16.9)	2.9 (0.1-16.4)	4.7 (0.1-37.9)
			Corynebacteriaceae	Corynebacterium	0.1954	**0.0043**	0.1 (0-1.7)	0.1 (0-1.2)	0.1 (0-0.8)	0.1 (0-1)	0.1 (0-2.1)	0.2 (0-3.9)	0.1 (0-1.7)
			Microbacteriaceae	Cryocola	0.7561	0.7710	0.1 (0-0.8)	0.2 (0-2.1)	0 (0-0.3)	0.1 (0-1)	0 (0-0.3)	0.1 (0-2.5)	0 (0-0.6)
				Other	0.0088	0.1069	0.1 (0-1.6)	0 (0-0.7)	0.1 (0-1)	0 (0-0.4)	0.2 (0-2.9)	0.1 (0-1)	0.4 (0-4)

Taxon					P-value		Sample type						
Phylum	Class	Order	Family	Genus	Breed	Environ-ment	Bengal	Cornish Rex	Devon Rex	Siberian	Sphynx	Indoor	Outdoor
			Micrococcaceae	Micrococcus	0.1583	0.0659	7.8 (0.3-37.1)	9.2 (0.2-47.2)	8.2 (0.4-22)	11.9 (1.4-30.7)	8 (0.6-26.9)	12.5 (0.1-38.6)	8.7 (0.5-65.1)
				Rothia	0.3893	0.0561	0.7 (0-5.7)	0.3 (0-1.6)	0.3 (0-4.4)	0.1 (0-0.7)	0.6 (0-7.3)	0.2 (0-2.4)	0.8 (0-8.3)
			Nocardioidaceae		0.0526	0.4145	0.1 (0-3.4)	0 (0-1.1)	0 (0-0.1)	0.1 (0-0.4)	0.2 (0-2.2)	0.3 (0-7.4)	0.6 (0-4.4)
			Pseudonocardiaceae	Pseudonocardia	0.4262	0.4960	1.9 (0-30)	2.1 (0-30.2)	0.5 (0-2.8)	0.5 (0-3.6)	1.5 (0-41.9)	2 (0-27.2)	0.8 (0-5.7)
			Streptomycetaceae	Streptomyces	**0.0147**	0.3352	0 (0-0.3)	0 (0-0.3)	0 (0-0)	0 (0-0.1)	0 (0-0.3)	0.1 (0-1.4)	0.1 (0-1.8)
	Bifidobacteriales	Bifidobacteriales	Bifidobacteriaceae	Bifidobacterium	**0.0002**	0.7460	0 (0-0.8)	0.1 (0-1.9)	0 (0-0)	0 (0-0.1)	0.1 (0-2.4)	0 (0-1)	0.2 (0-3.9)

Taxon					P-value		Sample type						
Phylum	Class	Order	Family	Genus	Breed	Environ-ment	Bengal	Cornish Rex	Devon Rex	Siberian	Sphynx	Indoor	Outdoor
	Coriobacteriia	Coriobacteriales	Coriobacteriaceae	Collinsella	**0.0106**	0.3760	0 (0-0.2)	0 (0-1.5)	0 (0-0)	0 (0-0.3)	0 (0-0.6)	0.1 (0-5)	0.1 (0-1.5)
	Rubrobacteria	Rubrobacterales	Rubrobacteraceae	Rubrobacter	**0.0029**	0.7473	8.2 (0.4-28.8)	7.1 (0.2-34.7)	6.3 (0.9-33.4)	5.5 (0.7-13.3)	7.9 (0.5-26.5)	6.4 (0.3-38)	8.6 (0.3-45.7)
	Thermoleophilia	Solirubrobacterales	Solirubrobacteraceae		0.5554	0.7554	3.7 (0.1-18.8)	3.8 (0.1-16.6)	3.4 (0.2-17.7)	3.8 (0.2-18.2)	3.7 (0.1-16.9)	2.9 (0.1-16.4)	4.7 (0.1-37.9)
Bacteroidetes	Saprospirae	Saprospirales	Chitinophagaceae		0.3251	0.4033	0 (0-0.2)	0 (0-0.2)	0 (0-0.1)	0 (0-0.2)	0 (0-0.5)	0 (0-1.8)	0.1 (0-1.4)
				Flavisolibacter	0.1876	0.3733	0.1 (0-1.7)	0.1 (0-1.2)	0.1 (0-0.8)	0.1 (0-1)	0.1 (0-2.1)	0.2 (0-3.9)	0.1 (0-1.7)
	Bacteroidia	Bacteroidales	Paraprevotellaceae	Prevotella	0.5741	0.6041	0.1 (0-3.9)	0 (0-1.7)	0 (0-0.6)	0 (0-0.3)	0.1 (0-1.5)	0.1 (0-5.5)	0.4 (0-5.2)

Taxon					P-value		Sample type						
Phylum	Class	Order	Family	Genus	Breed	Environ-ment	Bengal	Cornish Rex	Devon Rex	Siberian	Sphynx	Indoor	Outdoor
			Bacteroidaceae	Bacteroides	0.2341	0.6015	3.9 (0.2-46.6)	6.9 (0.1-66)	2.6 (0.2-18.9)	9.5 (0.1-83.2)	6.7 (0.1-86)	7.9 (0.2-92.9)	5 (0.1-30.6)
			Porphyromonadaceae	Paludibacter	0.1415	0.7717	0.3 (0-1.4)	0.3 (0-2.2)	0.1 (0-0.2)	0.5 (0-4.7)	0.5 (0-5.1)	0.2 (0-2.6)	0.6 (0-3.7)
				Porphyromonas	**0.0003**	0.9016	1.9 (0.1-20.9)	1.1 (0-7.5)	0.9 (0-6.7)	1 (0-6.4)	2.3 (0-22.1)	1.3 (0-16.4)	1.5 (0-8.4)
			Prevotellaceae	Prevotella	**0.0003**	0.1303	0.6 (0-8.1)	1.2 (0-4.9)	0.5 (0-3)	1.7 (0-8.5)	0.9 (0-12.8)	0.5 (0-6.6)	0.4 (0-2.5)
	Cytophagia	Cytophagales	Cytophagaceae	Adhaeribacter	0.3376	0.7434	0.1 (0-0.8)	0.2 (0-2.1)	0 (0-0.3)	0.1 (0-1)	0 (0-0.3)	0.1 (0-2.5)	0 (0-0.6)
	Flavobacteriia	Flavobacteriales	Weeksellaceae		0.1113	0.7214	0 (0-0.1)	0 (0-0)	0 (0-0)	0 (0-0)	0 (0-1)	0 (0-0)	0.1 (0-2.1)
				Cloacibacterium	0.3915	0.8984	0.1 (0-1.6)	0 (0-0.7)	0 (0-0.2)	0 (0-0)	0.2 (0-2.9)	0 (0-0.8)	0.2 (0-4)

Taxon					P-value		Sample type						
Phylum	Class	Order	Family	Genus	Breed	Environ-ment	Bengal	Cornish Rex	Devon Rex	Siberian	Sphynx	Indoor	Outdoor
			Flavobacteriaceae	Capnocytophaga	0.0149	0.3680	0.2 (0-2.7)	0.1 (0-1.5)	0.1 (0-0.6)	0.2 (0-1.8)	0.2 (0-2.1)	0.6 (0-8.6)	0.2 (0-3.5)
	Sphingobacteriia	Sphingobacteriales	Sphingobacteriaceae	Sphingobacterium	0.1053	0.9917	0.5 (0-9.3)	0 (0-0.6)	0.4 (0-1.4)	0.4 (0-5.1)	0.5 (0-11.6)	0.4 (0-7.4)	0.7 (0-6.1)
Chlorobi	OPB56				0.1878	0.3641	7.7 (0.3-37)	9 (0.2-44.2)	7.3 (0.4-22)	11.6 (1.4-30.7)	6.8 (0.3-26.8)	12.2 (0.1-36.2)	8.5 (0.5-65.1)
Firmicutes	Bacilli	Bacillales			0.1880	0.8464	0.1 (0-2.4)	0 (0-1.1)	0 (0-0.1)	0 (0-0.4)	0.2 (0-2.2)	0.3 (0-7.4)	0.5 (0-3.5)
			Bacillaceae		**0.0004**	0.7295	0.4 (0-5.4)	0.1 (0-0.5)	0 (0-0.5)	0.1 (0-0.5)	0.4 (0-12.3)	0.8 (0-27)	0.4 (0-4.5)
				Bacillus	0.0957	0.7483	1.9 (0-30)	2.1 (0-30.2)	0.5 (0-2.8)	0.5 (0-3.6)	1.5 (0-41.9)	2 (0-27.2)	0.8 (0-5.7)
				Geobacillus	0.3650	0.6103	0.3 (0-12.1)	4.6 (0-31.9)	0.9 (0-9.4)	0.5 (0-6)	0.1 (0-1.2)	0.1 (0-1.8)	0.1 (0-0.6)
			Planococcaceae		0.1055	0.4833	0 (0-0.8)	0.1 (0-0.9)	0 (0-0.1)	0 (0-0.2)	0.1 (0-1.6)	0.1 (0-4.1)	0 (0-0.7)
				Sporosarcina	**<0.0001**	0.3249	0 (0-0.8)	0.1 (0-1.9)	0 (0-0)	0 (0-0.1)	0.1 (0-2.4)	0 (0-1)	0.2 (0-3.9)
			Staphylococcaceae	Jeotgalicoccus	0.0647	0.7721	0 (0-0.1)	0 (0-1.5)	0 (0-0)	0 (0-0.3)	0 (0-0.6)	0.1 (0-5)	0.1 (0-1.5)

Taxon					P-value		Sample type						
Phylum	Class	Order	Family	Genus	Breed	Environ-ment	Bengal	Cornish Rex	Devon Rex	Siberian	Sphynx	Indoor	Outdoor
				Staphylococcus	0.3159	0.7741	0.1 (0-1.1)	0 (0-0.3)	0 (0-0.3)	0.2 (0-1.8)	0.2 (0-3.8)	0.1 (0-1.8)	0 (0-0.7)
		Lactobacillales	Aerococcaceae		**0.0100**	0.7491	0 (0-0.2)	0.1 (0-0.5)	0 (0-0.5)	0.1 (0-1.5)	0.1 (0-1.1)	0.1 (0-0.9)	0.1 (0-1.1)
			Enterococcaceae	Enterococcus	0.0729	0.7520	0.1 (0-2.2)	0 (0-1.7)	0 (0-0.6)	0 (0-0.3)	0.1 (0-1.1)	0 (0-0.3)	0.3 (0-4)
			Lactobacillaceae	Lactobacillus	**<0.0001**	0.7196	0.3 (0-8.2)	0.4 (0-4.5)	0.1 (0-0.7)	0.2 (0-1.5)	0.2 (0-5.3)	0.4 (0-7.2)	0.1 (0-1.1)
			Streptococcaceae	Streptococcus	**0.0074**	0.7986	0.4 (0-3.3)	0.2 (0-1.5)	0.8 (0-11.3)	0.2 (0-1.1)	0.3 (0-2.1)	0.7 (0-7)	0.5 (0-3.9)
			Other	Other	0.0973	0.3742	0.2 (0-1.7)	3 (0-22.5)	0.3 (0-1.9)	0.2 (0-0.8)	0.3 (0-1.9)	0.3 (0-6)	0.6 (0-7.1)

Taxon					P-value		Sample type						
Phylum	Class	Order	Family	Genus	Breed	Environ-ment	Bengal	Cornish Rex	Devon Rex	Siberian	Sphynx	Indoor	Outdoor
	Clostridia	Clostridiales			0.1394	0.9228	1.3 (0-15.2)	0.2 (0-1.7)	0.2 (0-1)	0.5 (0-2.5)	0.8 (0-6.8)	1.5 (0-12.2)	1.2 (0-12.4)
			Acidaminobacteraceae	Fusibacter	0.0722	0.5523	0 (0-0.1)	0 (0-0)	0 (0-0)	0 (0-0)	0 (0-0.1)	0 (0-0)	0.1 (0-1.3)
			Tissierellaceae	Parvimonas	0.3897	0.2270	0.8 (0-9.7)	0.2 (0-2.5)	0.5 (0-1.4)	0.6 (0-5.6)	0.7 (0-11.6)	0.5 (0-7.4)	1.7 (0-18.5)
			Clostridiaceae		**<0.0001**	0.0517	9 (0.3-37.2)	9.7 (0.3-47.4)	8.6 (0.5-22.5)	12.4 (1.9-32.1)	9 (0.7-27.5)	13.2 (0.2-38.6)	10.6 (0.6-65.2)
				Clostridium	**0.0003**	0.1172	17 (0.8-49.8)	26 (0.4-62.1)	10.9 (2.4-24.7)	16.1 (2.1-44.5)	14.7 (0.9-51.8)	16.2 (0.5-58.2)	14 (0.6-40)
			Lachnospiraceae		0.1578	0.7787	0.3 (0-3.9)	0.2 (0-2.9)	0.1 (0-0.6)	0.7 (0-4.3)	0.1 (0-0.8)	0.2 (0-2)	0.2 (0-2)
				Ruminococcus	0.0010	0.5449	0.2 (0-2.7)	0.3 (0-2)	0.1 (0-1.5)	0.1 (0-0.6)	0.4 (0-4.2)	0.4 (0-5)	1.1 (0-8.2)
				Blautia	**<0.0001**	0.1946	23.2 (3.3-75.2)	15.7 (0.6-46.5)	15.4 (1.8-50.3)	17.1 (2.6-48.2)	26 (2.4-85.4)	20.4 (0.9-97.5)	21.9 (2-71.8)
				Other	0.1888	0.4973	0.2 (0-4.9)	0.1 (0-1.7)	0 (0-0.6)	0 (0-0.4)	0.2 (0-1.7)	0.2 (0-5.5)	0.5 (0-6.5)
			Peptococcaceae	Peptococcus	0.3438	0.3400	43.7 (8.5-76.8)	41.7 (7.1-98.5)	59.9 (24.6-90.9)	44.9 (12.4-88.5)	42.3 (7.3-89.4)	43.3 (1.4-97)	43.2 (13-92.2)

Taxon					P-value		Sample type						
Phylum	Class	Order	Family	Genus	Breed	Environ-ment	Bengal	Cornish Rex	Devon Rex	Siberian	Sphynx	Indoor	Outdoor
			Peptostreptococcaceae		**0.0477**	0.9783	0.6 (0-8.1)	1.2 (0-4.9)	0.5 (0-3)	1.7 (0-8.5)	0.9 (0-12.9)	0.5 (0-6.6)	0.5 (0-2.5)
				Filifactor	**0.0190**	0.7937	0.6 (0-9.9)	0.1 (0-0.6)	0.1 (0-0.8)	0.2 (0-2.1)	0.4 (0-6)	0.7 (0-12.9)	0.4 (0-3.3)
			Ruminococcaceae		0.0529	0.3857	0.1 (0-0.8)	0.2 (0-2.1)	0 (0-0.3)	0.1 (0-1)	0 (0-0.3)	0.1 (0-2.5)	0 (0-0.6)
			Veillonellaceae	Megamonas	**0.0079**	0.4475	0.2 (0-2.6)	0.2 (0-2.1)	0 (0-0.5)	0.1 (0-1.2)	0.1 (0-3.2)	0 (0-0.7)	0.5 (0-11.1)
				Megasphaera	**<0.0001**	0.1501	0.5 (0-9.3)	0 (0-0.6)	0.4 (0-1.4)	0.4 (0-5.1)	0.5 (0-11.6)	0.4 (0-7.4)	0.7 (0-6.1)
				Veillonella	0.3405	0.7380	0.7 (0-5.7)	0.3 (0-1.6)	0.3 (0-4.4)	0.1 (0-0.7)	0.6 (0-7.3)	0.2 (0-2.4)	0.8 (0-8.3)
Fusobacteria	Fusobacteriia	Fusobacteriales	Fusobacteriaceae	Fusobacterium	**0.0237**	0.0991	0.3 (0-7.9)	0.2 (0-2.4)	0.1 (0-0.6)	0.3 (0-5.4)	0.1 (0-1)	0.1 (0-1.8)	0 (0-0.3)
			Leptotrichiaceae		0.1170	0.1979	0 (0-0.9)	0 (0-0)	0 (0-0.1)	0.1 (0-1)	0 (0-0.9)	0.1 (0-2.5)	0.3 (0-3.2)
				Other	0.1114	0.7550	0.5 (0-7.7)	0.1 (0-0.5)	0.3 (0-1.6)	0.3 (0-2.3)	1 (0-23.5)	0.9 (0-29)	0.7 (0-15.1)

Taxon					P-value		Sample type						
Phylum	Class	Order	Family	Genus	Breed	Environ-ment	Bengal	Cornish Rex	Devon Rex	Siberian	Sphynx	Indoor	Outdoor
Proteobacteria	Alphaproteobacteria	Caulobacterales	Caulobacteraceae		0.4821	0.9959	1.9 (0-30)	2.1 (0-30.2)	0.5 (0-2.8)	0.5 (0-3.6)	1.5 (0-41.9)	2 (0-27.2)	0.8 (0-5.7)
		Rhizobiales	Bradyrhizobiaceae	Bradyrhizobium	0.7002	0.7279	0 (0-0.3)	0 (0-0.6)	0 (0-0)	0 (0-0.6)	0.1 (0-2.2)	0.1 (0-1.1)	0.3 (0-3)
			Methylobacteriaceae	Methylobacterium	0.0734	0.6048	14.9 (0.5-72.7)	8.5 (0.4-32.6)	9 (0.9-22.9)	11.6 (0.4-45.5)	18 (0.8-83.2)	13.9 (0.3-97)	13.2 (0.9-66)
			Phyllobacteriaceae		**0.0103**	0.6097	8.2 (0.4-28.8)	7.1 (0.2-34.7)	6.3 (0.9-33.4)	5.5 (0.7-13.3)	7.9 (0.5-26.5)	6.4 (0.3-38)	8.6 (0.3-45.7)
				Phyllobacterium	0.6666	0.8235	0 (0-0.2)	0.1 (0-0.5)	0 (0-0.5)	0.1 (0-1.5)	0.1 (0-1.1)	0.1 (0-0.9)	0.1 (0-1.1)
		Rhodobacterales	Rhodobacteraceae		0.1756	0.7603	0 (0-0.2)	0 (0-0.2)	0 (0-0.1)	0 (0-0.2)	0 (0-0.5)	0 (0-1.8)	0.1 (0-1.4)
				Paracoccus	0.2732	0.7788	0.1 (0-1.7)	0.1 (0-1.2)	0.1 (0-0.8)	0.1 (0-1)	0.1 (0-2.1)	0.2 (0-3.9)	0.1 (0-1.7)

Taxon					P-value		Sample type						
Phylum	Class	Order	Family	Genus	Breed	Environ-ment	Bengal	Cornish Rex	Devon Rex	Siberian	Sphynx	Indoor	Outdoor
		Rhodospirillales	Acetobacteraceae		**0.0100**	0.3738	3.9 (0.2-46.6)	6.9 (0.1-66)	2.6 (0.2-18.9)	9.5 (0.1-83.2)	6.7 (0.1-86)	7.9 (0.2-92.9)	5 (0.1-30.6)
		Sphingomonadaceae	Sphingomonadaceae		**0.0240**	0.7521	1.9 (0.1-20.9)	1.1 (0-7.5)	0.9 (0-6.7)	1 (0-6.4)	2.3 (0-22.1)	1.3 (0-16.4)	1.5 (0-8.4)
				Sphingomonas	0.0504	0.9843	0.6 (0-9.9)	0.1 (0-0.6)	0.1 (0-0.8)	0.2 (0-2.1)	0.4 (0-6)	0.7 (0-12.9)	0.4 (0-3.3)
	Betaproteobacteria	Burkholderiales	Alcaligenaceae	Sutterella	**<0.0001**	0.9573	0.2 (0-2.7)	0.1 (0-1.5)	0.1 (0-0.6)	0.2 (0-1.8)	0.2 (0-2.1)	0.6 (0-8.6)	0.2 (0-3.5)
			Burkholderiaceae	Lautropia	0.5172	0.5193	0.5 (0-9.3)	0 (0-0.6)	0.4 (0-1.4)	0.4 (0-5.1)	0.5 (0-11.6)	0.4 (0-7.4)	0.7 (0-6.1)
			Comamonadaceae		0.6615	0.7546	7.7 (0.3-37)	9 (0.2-44.2)	7.3 (0.4-22)	11.6 (1.4-30.7)	6.8 (0.3-26.8)	12.2 (0.1-36.2)	8.5 (0.5-65.1)
				Acidovorax	0.3180	0.7445	0.2 (0-3.9)	0.2 (0-3)	0.9 (0-15.8)	0.2 (0-2.5)	1.2 (0-12.1)	0.3 (0-3.6)	0.2 (0-1.7)
				Lampropedia	0.1730	0.9038	0.7 (0-5.7)	0.3 (0-1.6)	0.3 (0-4.4)	0.1 (0-0.7)	0.6 (0-7.3)	0.2 (0-2.4)	0.8 (0-8.3)
				Other	0.4444	0.9955	0 (0-0.9)	0 (0-0)	0 (0-0.1)	0.1 (0-1)	0 (0-0.9)	0.1 (0-2.5)	0.3 (0-3.2)

Taxon					P-value		Sample type						
Phylum	Class	Order	Family	Genus	Breed	Environ-ment	Bengal	Cornish Rex	Devon Rex	Siberian	Sphynx	Indoor	Outdoor
			Oxalobacteraceae		**0.0015**	0.4690	0.1 (0-2.4)	0 (0-1.1)	0 (0-0.1)	0 (0-0.4)	0.2 (0-2.2)	0.3 (0-7.4)	0.5 (0-3.5)
		Neisseriales	Neisseriaceae		**<0.0001**	0.7428	1.9 (0-30)	2.1 (0-30.2)	0.5 (0-2.8)	0.5 (0-3.6)	1.5 (0-41.9)	2 (0-27.2)	0.8 (0-5.7)
				Conchiformibius	**0.0004**	0.1355	0.3 (0-12.1)	4.6 (0-31.9)	0.9 (0-9.4)	0.5 (0-6)	0.1 (0-1.2)	0.1 (0-1.8)	0.1 (0-0.6)
				Other	0.0741	0.5908	0 (0-0.8)	0.1 (0-0.9)	0 (0-0.1)	0 (0-0.2)	0.1 (0-1.6)	0.1 (0-4.1)	0 (0-0.7)
		Other	Other	Other	**0.0003**	0.9368	9.4 (0.2-69.2)	3.7 (0.2-18.3)	4.4 (0.2-14.3)	7.7 (0.3-44)	10.6 (0.3-80.8)	10.1 (0.2-96.2)	8.9 (0.3-65.4)
	Epsilonproteobacteria	Campylobacterales	Campylobacteraceae	Arcobacter	**<0.001**	0.4275	3.7 (0.1-18.8)	3.8 (0.1-16.6)	3.4 (0.2-17.7)	3.8 (0.2-18.2)	3.7 (0.1-16.9)	2.9 (0.1-16.4)	4.7 (0.1-37.9)
				Campylobacter	0.0539	0.7412	0.1 (0-1.7)	0.1 (0-1.2)	0.1 (0-0.8)	0.1 (0-1)	0.1 (0-2.1)	0.2 (0-3.9)	0.1 (0-1.7)
			Helicobacteraceae	Flexispira	0.1717	0.7036	0.1 (0-2.2)	0 (0-1.7)	0 (0-0.6)	0 (0-0.3)	0.1 (0-1.1)	0 (0-0.3)	0.3 (0-4)
				Helicobacter	**0.0041**	0.4905	0.3 (0-8.2)	0.4 (0-4.5)	0.1 (0-0.7)	0.2 (0-1.5)	0.2 (0-5.3)	0.4 (0-7.2)	0.1 (0-1.1)
	Gammaproteobacteria	Cardiobacteriales	Cardiobacteriaceae		0.3025	0.9993	1.3 (0-15.2)	0.2 (0-1.7)	0.2 (0-1)	0.5 (0-2.5)	0.8 (0-6.8)	1.5 (0-12.2)	1.2 (0-12.4)

Taxon					P-value		Sample type						
Phylum	Class	Order	Family	Genus	Breed	Environ-ment	Bengal	Cornish Rex	Devon Rex	Siberian	Sphynx	Indoor	Outdoor
		Enterobacteriales	Enterobacteriaceae		**0.0028**	0.6890	Bengal	Cornish Rex	Devon Rex	Siberian	Sphynx	Indoor	Outdoor
		Pasteurellales	Pasteurellaceae		**0.0277**	0.7337	23.2 (3.3-75.2)	15.7 (0.6-46.5)	15.4 (1.8-50.3)	17.1 (2.6-48.2)	26 (2.4-85.4)	20.4 (0.9-97.5)	21.9 (2-71.8)
				Aggregatibacter	0.0723	0.3748	0 (0-0.7)	0 (0-0.2)	0 (0-0.1)	0 (0-0.4)	0.1 (0-1.3)	0.1 (0-1.8)	0.2 (0-1.9)
				Haemophilus	0.1293	0.6177	0.1 (0-1.7)	0.1 (0-1.2)	0.1 (0-0.8)	0.1 (0-1)	0.1 (0-2.1)	0.2 (0-3.9)	0.1 (0-1.7)
				Pasteurella	0.4416	0.1904	0.2 (0-4.9)	0.1 (0-1.7)	0 (0-0.6)	0 (0-0.4)	0.2 (0-1.7)	0.2 (0-5.5)	0.5 (0-6.5)
		Pseudomonadales	Moraxellaceae		**<0.0001**	0.3694	43.7 (8.5-76.8)	41.7 (7.1-98.5)	59.9 (24.6-90.9)	44.9 (12.4-88.5)	42.3 (7.3-89.4)	43.3 (1.4-97)	43.2 (13-92.2)
				Acinetobacter	**<0.0001**	0.7615	0.6 (0-8.1)	1.2 (0-4.9)	0.5 (0-3)	1.7 (0-8.5)	0.9 (0-12.9)	0.5 (0-6.6)	0.5 (0-2.5)
				Enhydrobacter	**0.0078**	0.8193	0.6 (0-9.9)	0.1 (0-0.6)	0.1 (0-0.8)	0.2 (0-2.1)	0.4 (0-6)	0.7 (0-12.9)	0.4 (0-3.3)
				Moraxella	0.6662	0.1947	0.1 (0-0.8)	0.2 (0-2.1)	0 (0-0.3)	0.1 (0-1)	0 (0-0.3)	0.1 (0-2.5)	0 (0-0.6)
			Pseudomonadaceae		0.1348	0.7508	0.1 (0-1.6)	0 (0-0.7)	0.1 (0-1)	0 (0-0.4)	0.2 (0-2.9)	0.1 (0-1)	0.4 (0-4)
				Pseudomonas	0.3212	0.7616	0.2 (0-2.7)	0.1 (0-1.5)	0.1 (0-0.6)	0.2 (0-1.8)	0.2 (0-2.1)	0.8 (0-11)	0.2 (0-3.5)
		Xanthomonadales	Xanthomonadaceae		**0.0295**	0.4996	0.2 (0-2.6)	0.2 (0-2.1)	0 (0-0.5)	0.1 (0-1.2)	0.1 (0-3.2)	0 (0-0.7)	0.5 (0-11.1)

Taxon					P-value		Sample type						
Phylum	Class	Order	Family	Genus	Breed	Environ-ment	Bengal	Cornish Rex	Devon Rex	Siberian	Sphynx	Indoor	Outdoor
				Luteimonas	0.0726	0.6181	0.5 (0-9.3)	0 (0-0.6)	0.4 (0-1.4)	0.4 (0-5.1)	0.5 (0-11.6)	0.4 (0-7.4)	0.7 (0-6.1)
Spirochaetes	Spirochaetes	Spirochaetales	Spirochaetaceae	Treponema	0.0572	0.6046	0.7 (0-5.7)	0.3 (0-1.6)	0.3 (0-4.4)	0.1 (0-0.7)	0.6 (0-7.3)	0.2 (0-2.4)	0.8 (0-8.3)
SR1					0.0878	0.7876	0.3 (0-7.9)	0.2 (0-2.4)	0.1 (0-0.6)	0.3 (0-5.4)	0.1 (0-1)	0.1 (0-1.8)	0 (0-0.3)
Tenericutes	Mollicutes	Acholeplasmatales	Acholplasmataceae	Acholeplasma	0.1893	0.3537	0.1 (0-3.4)	0 (0-1.1)	0 (0-0.1)	0.1 (0-0.4)	0.2 (0-2.2)	0.3 (0-7.4)	0.6 (0-4.4)
