# Supplementary material for: The feline cutaneous and oral microbiota are influenced by breed and environment
Source: PLoS One. 2019 Jul 30;14(7):e0220463. doi: 10.1371/journal.pone.0220463 (PMC6667137; doi:10.1371/journal.pone.0220463)
Supplement: S4 Table — Average (min-max). P<0.05 are bolded. (PDF) [file pone.0220463.s011.pdf]

Table S4. Relative abundance of fungal genera present at 1% in at least 10 samples. Average (min-max). P<0.05 are bolded.

Taxon					p-value		Sample type						
Phylum	Class	Order	Family	Genus	Breed	Environ-ment	Bengal	Cornish Rex	Devon Rex	Siberian	Sphynx	Indoor	Outdoor
Ascomycota	Ascomycota class incertae sedis	Ascomycota order incertae sedis	Ascomycota family incertae sedis	Thermomyces	**0.0051**	1.0331	0.7 (0-34.8)	0 (0-0.4)	0 (0-0.1)	0 (0-0.2)	2 (0-32.8)	0.5 (0-28.9)	0.1 (0-1.8)
	Dothideomycetes	Capnodiales	Capnodiales family incertae sedis	Cladosporium	**0.0009**	0.9761	5.4 (0.2-30.7)	12.8 (0.1-84.3)	3.9 (0.1-27.8)	6.1 (0.1-50.9)	8.6 (0.1-67.8)	23.4 (0.1-96.9)	25.9 (0.1-97.3)
			unclassified Capnodiales family|	unclassified Capnodiales genus	0.6370	0.9063	0.5 (0-18.3)	0.1 (0-2.1)	0.8 (0-7.5)	5.8 (0-93.2)	0.1 (0-2.5)	0 (0-0.4)	0.2 (0-4.7)
			Dothioraceae	unclassified Dothioraceae genus	0.1859	1.0604	0.3 (0-5.7)	2.6 (0-81.9)	0.3 (0-4.1)	0.7 (0-10.3)	1.1 (0-17.7)	1.8 (0-51.8)	0.4 (0-12.6)
		Pleosporales	Pleosporaceae	Alternaria	**0.0064**	1.0030	1.6 (0-26.9)	0.1 (0-3.2)	0.7 (0-13.3)	0.1 (0-1.2)	3.9 (0-94.2)	0.4 (0-10.8)	0.6 (0-5.8)
				Other	**0.0086**	0.9593	2.6 (0-31.5)	1.9 (0-42.6)	2.9 (0-30.2)	3.1 (0-39.9)	3.5 (0-33.9)	5 (0-73.9)	0.5 (0-4.2)

Taxon					p-value		Sample type						
Phylum	Class	Order	Family	Genus	Breed	Environ-ment	Bengal	Cornish Rex	Devon Rex	Siberian	Sphynx	Indoor	Outdoor
				unclassified Pleosporaceae genus	**<0.0001**	0.8535	1.9 (0-26.8)	2.5 (0-37.1)	0.2 (0-0.9)	5 (0-69.5)	11.3 (0-77.4)	2 (0-44)	5.9 (0-95.9)
			Pleosporales family incertae sedis	Leptosphaerulina	0.4270	0.9483	12.1 (0-90.1)	0.3 (0-5.9)	3.2 (0-55.7)	0.5 (0-9.2)	8.9 (0-74.2)	1.7 (0-81.5)	3.6 (0-49.5)
			unclassified Pleosporales family	unclassified Pleosporales genus	0.1099	0.8909	2.6 (0-44.2)	3.8 (0-55.2)	0.5 (0-5.4)	5.7 (0-92)	2.9 (0-48.1)	4.3 (0-96.3)	8.6 (0-82.6)
	Eurotiomycetes	Eurotiales	Trichocomaceae	Aspergillus	**0.0026**	0.9543	7 (0-77.1)	1.1 (0-24.6)	6.4 (0-97.7)	0.8 (0-11.7)	3.4 (0-94.2)	7.7 (0-98.1)	1.4 (0-15.4)
				Other	**<0.0001**	0.9087	3.2 (0-83.2)	2.7 (0-64.9)	0 (0-0.2)	0.2 (0-3.6)	2.4 (0-59.9)	0.8 (0-42)	0.2 (0-3.3)
				Penicillium	0.0712	1.2943	1.1 (0-13.3)	5.1 (0-95.1)	2.4 (0-37.7)	5 (0-93.7)	5.3 (0-98.3)	3 (0-90.4)	3 (0-78.4)
	Other	Other	Other	Other	0.1201	0.9434	0.3 (0-3.8)	0 (0-0.7)	0 (0-0.2)	0 (0-0.1)	0.1 (0-0.7)	1.9 (0-75.3)	0.1 (0-1.6)
	Saccharomycetes	Saccharomycetales	Saccharomycetaceae	Saccharomyces	0.1055	0.9235	0.6 (0-12.7)	0.4 (0-5.6)	0.2 (0-3.2)	0.1 (0-0.6)	0.8 (0-45.6)	0.4 (0-10.4)	0.3 (0-9.4)

Taxon					p-value		Sample type						
Phylum	Class	Order	Family	Genus	Breed	Environ-ment	Bengal	Cornish Rex	Devon Rex	Siberian	Sphynx	Indoor	Outdoor
			Saccharomycetales family incertae sedis	Candida	**0.0136**	0.9470	0.2 (0-2.6)	0.2 (0-3.6)	0.1 (0-0.9)	0.1 (0-0.9)	1.1 (0-28.9)	0.1 (0-2.6)	0 (0-0.5)
				Debaryomyces	0.5947	0.8895	2.8 (0-67.8)	15.6 (0-91.3)	0.1 (0-0.3)	1.6 (0-45.9)	0.1 (0-1.1)	0.3 (0-10)	2.3 (0-88.7)
			unclassified Saccharomycetales famil	unclassified Saccharomycetales genus	0.0955	0.9248	0.5 (0-15.6)	0.4 (0-13.6)	5.2 (0-97.7)	0.1 (0-1.1)	0.2 (0-2.2)	0 (0-0.3)	0 (0-0.3)
	Sordariomycetes	Hypocreales	Hypocreales family incertae sedis	Fusarium	0.0557	1.2009	4.2 (0-42.3)	0.6 (0-10.1)	4.5 (0-71.7)	3.4 (0-87.3)	3 (0-24.4)	6.5 (0-40.6)	7.6 (0-91.4)
				Myrothecium	0.0905	1.6716	0.3 (0-6.8)	1.8 (0-47.7)	0 (0-0.1)	0.2 (0-4.8)	0.3 (0-9)	0.3 (0-9.8)	0.3 (0-10)
				Sarocladium	**0.0290**	1.0319	0.2 (0-2.2)	2.6 (0-98)	0 (0-0.1)	1.7 (0-49.4)	0.9 (0-21.5)	0.3 (0-3.6)	0.1 (0-2.2)
			Other	Other	0.1156	0.8520	0.2 (0-4)	0.1 (0-1.5)	0 (0-0.1)	0.7 (0-20.8)	0.3 (0-5.5)	0.4 (0-17.8)	0.4 (0-14.4)
			unclassified Hypocreales family	unclassified Hypocreales genus	**0.0051**	1.0046	0.2 (0-7.1)	0 (0-0.6)	0 (0-0.2)	0 (0-0.3)	1.1 (0-38.7)	0.2 (0-2.9)	0.1 (0-1.6)

Taxon					p-value		Sample type						
Phylum	Class	Order	Family	Genus	Breed	Environ-ment	Bengal	Cornish Rex	Devon Rex	Siberian	Sphynx	Indoor	Outdoor
		Trichosphaeriales	Trichosphaeriales family incertae sedis	Nigrospora	**0.0075**	0.9847	1.8 (0-36.1)	0.2 (0-3.3)	0.1 (0-1.1)	5.4 (0-88.3)	1.9 (0-46.6)	7.5 (0-97.4)	3.3 (0-41.9)
	unclassified Ascomycota class	unclassified Ascomycota order	unclassified Ascomycota family	unclassified Ascomycota genus	**0.0189**	0.8147	0.4 (0-9.1)	0.2 (0-6.4)	2 (0-11)	0.1 (0-0.9)	1.7 (0-66.5)	2.5 (0-97.7)	0.4 (0-7.3)
Basidiomycota	Agaricomycetes	Agaricomycetes order incertae sedis	Corticiaceae	Phanerochaete	0.8788	1.0515	0.2 (0-5.4)	0.3 (0-9.3)	0.1 (0-1.5)	0 (0-0.3)	0 (0-0.2)	1.8 (0-93.5)	0.1 (0-2.9)
			Peniophoraceae	Peniophora	0.4179	0.9887	0.5 (0-14.1)	1 (0-44.6)	0.5 (0-8.7)	1.9 (0-50.6)	0.5 (0-25.4)	0.9 (0-25.8)	0.1 (0-1.3)
			Stereaceae	Stereum	**0.0034**	1.0299	1.1 (0-26.5)	2.9 (0-83.1)	4 (0-74.7)	0.4 (0-7.3)	0.1 (0-2.1)	0.1 (0-1.6)	0 (0-0.3)
		Polyporales	Polyporales family incertae sedis	Fomes	**0.0349**	0.9736	0.3 (0-4.3)	0.1 (0-0.5)	0.9 (0-16.3)	0.2 (0-2.3)	0.1 (0-1)	2 (0-96.5)	0.9 (0-32.6)
				Gelatoporia	0.3585	1.4040	0.9 (0-34.5)	0.1 (0-1.3)	1.5 (0-20.3)	0 (0-0.1)	0.4 (0-20.4)	0 (0-0.6)	0.1 (0-1.6)

Taxon					p-value		Sample type						
Phylum	Class	Order	Family	Genus	Breed	Environ-ment	Bengal	Cornish Rex	Devon Rex	Siberian	Sphynx	Indoor	Outdoor
				Trametes	0.5798	1.0264	0.5 (0-11.9)	1.5 (0-30.3)	2.4 (0-41.8)	0 (0-0.1)	0.5 (0-27.1)	0 (0-0.9)	0.2 (0-6.4)
	Agaricostilbomycetes	Agaricostilbomycetes order incertae sedis	Agaricostilbomycetes family incertae sedis	Sterigmatomyces	0.0926	0.5152	0.2 (0-7.9)	0.3 (0-12.9)	3.5 (0-45.7)	0 (0-0.1)	0 (0-2.1)	0 (0-0.2)	0.6 (0-10.1)
	Basidiomycota class incertae sedis	Basidiomycota class incertae sedis	Basidiomycota family incertae sedis	Wallemia	0.3670	1.2529	0.1 (0-2.2)	0.9 (0-38.3)	0.1 (0-2.4)	0.1 (0-1.9)	0.7 (0-24.8)	0.1 (0-2.5)	0.2 (0-4.6)
		Malasseziales	Malasseziales family incertae sedis	Malassezia	**0.0026**	0.9414	1.3 (0-12.5)	5.6 (0-98.4)	17.2 (0.1-98.2)	11.2 (0-94.4)	2.6 (0-59.7)	0.8 (0-9.5)	5.2 (0-96.9)
		Sporidiobolales	unclassified Sporidiobolales family	unclassified Sporidiobolales genus	**0.0006**	0.8599	0.3 (0-6.8)	0.4 (0-17.2)	0.1 (0-2.3)	0.1 (0-2.1)	1.2 (0-19.3)	0 (0-0.4)	0.3 (0-10.7)
	Tremellomycetes	Tremellales	Tremellales family incertae sedis	Cryptococcus	0.6497	1.0284	0.2 (0-5.4)	0.8 (0-9.9)	0.1 (0-1.9)	0.6 (0-12.8)	0.7 (0-29.6)	0.1 (0-1.4)	1.1 (0-46)

Taxon					p-value		Sample type						
Phylum	Class	Order	Family	Genus	Breed	Environ-ment	Bengal	Cornish Rex	Devon Rex	Siberian	Sphynx	Indoor	Outdoor
Fungi phylum incertae sedis	Fungi class incertae sedis	Mucorales	Choanephoraceae	unclassified Choanephoraceae genus	0.1253	0.9444	0.9 (0-38.2)	0 (0-0.3)	2.6 (0-34.5)	0 (0-0.1)	0.2 (0-7.7)	0.2 (0-5.6)	0.3 (0-9.1)
Other	Other	Other	Other	Other	0.0057	1.1681	32.2 (0.5-95.2)	16.6 (0.5-98.5)	21.3 (0.7-80.2)	32.3 (0.5-98.5)	17.3 (0.5-93.7)	12.5 (0.4-99.1)	15.5 (0.5-98.9)
