# Supplementary material for: The feline cutaneous and oral microbiota are influenced by breed and environment
Source: PLoS One. 2019 Jul 30;14(7):e0220463. doi: 10.1371/journal.pone.0220463 (PMC6667137; doi:10.1371/journal.pone.0220463)
Supplement: S5 Table — Average, median (min-max). (PDF) [file pone.0220463.s012.pdf]

Table S5. Relative abundance of *Malassezia* species. Average, median (min-max).

Malassezia species	Bengal	Cornish Rex	Devon Rex	Siberian	Sphynx	Indoor	Outdoor
M. dermatis	0.4, 0 (0-5.2)	0.2, 0 (0-7.1)	0.1, 1.0 (0-1.3)	0.1, 0 (0-1.8)	2.9, 0 (0-73.1)	0.3, 0 (0-10.0)	0.6, 0 (0-14.3)
M. furfur	6.9, 0 (0-40.6)	4.3, 0 (0-25.0)	0.2, 0 (0-3.7)	4.0, 0 (0-34.0)	13.9, 3.0 (0-84.8)	5.3, 0 (0-40.0)	6.9, 0 (0-60.0)
M. globosa	22.5, 17.7 (0-94.3)	22.7, 17.0 (0-80.0)	34.2, 23.9 (0-100.0)	26.0, 16.9 (0-96.2)	18.1, 12.1 (0-96.5)	26.3, 20.0 (0-99.0)	25.9, 23.1 (0-82.2)
M. japonica	0, 0 (0-0.3)	0, 0 (0-0)	0, 0 (0-0)	0, 0 (0-0.6)	0, 0 (0-0.4)	0, 0 (0-0)	0.1, 0 (0-6.7)
M. nana	5.3, 0 (0-90.9)	0.5, 0 (0-7.8)	4.6, 0 (0-59.5)	0.4, 0 (0-3.2)	10.9, 0.5 (0-88.6)	2.8, 0 (0-100.0)	1.1, 0 (0-16.7)
M. obtusa	0, 0 (0-0)	0, 0 (0-0)	0, 0 (0-0)	0, 0 (0-0)	0, 0 (0-0.1)	0, 0 (0-0)	0, 0 (0-0)
M. pachydermatis	0.7,0 (0-30.2)	0.7, 0 (0-21.9)	1.4, 0 (0-14.1)	0.2, 0 (0-4.3)	1.3, 0 (0-63.3)	2.4, 0 (0-46.2)	1.2, 0 (0-33.3)
M. restricta	32.5, 23.8 (2.2-93.9)	31.9, 25.1 (0-98.0)	42.3, 36.8 (0-96.8)	52.3, 52.9 (0.9-99.8)	31.1, 13.9 (0-100.0)	40.7, 34.7 (0-100.0)	38.8, 25.0 (0-99.4)
M. slooffiae	8.1. 0.1 (0-58.8)	18.5, 0 (0-100.0)	8.0, 0 (0-64.0)	5.0, 0 (0-63.6)	6.5, 0 (0-89.0)	4.9, 0 (0-66.7)	9.8, 0 (0-89.4)
M. sympodialis	0.5,0 (0-25.5)	0.3, 0 (0-8.3)	0.1, 0 (0-1.3)	0, 0 (0-0)	0, 0 (0-0.9)	0, 0 (0-0.2)	0.1, 0 (0-3.2)
Unclassified *Malassezia*	23.0, 7.6 (0-92.1)	21.1, 8.3 (0-88.5)	9.0, 4.0 (0-46.7)	12.0, 4.7 (0-96.8)	15.1, 6.1 (0-96.8)	17.2, 9.5 (0-100.0)	15.5, 4.9 (0-66.7)
