# Supplementary material for: The feline cutaneous and oral microbiota are influenced by breed and environment
Source: PLoS One. 2019 Jul 30;14(7):e0220463. doi: 10.1371/journal.pone.0220463 (PMC6667137; doi:10.1371/journal.pone.0220463)
Supplement: S6 Table — (PDF) [file pone.0220463.s013.pdf]

Table S6. Taxa determined to be differentially abundant on the skin across age groups and sex with LEfSe (LDA>2.5, p<0.01).

	**Bacteria**			**Fungi**		
	**Taxa**	**Group**	**LDA score**	**Taxa**	**Group**	**LDA score**
**Sex**	Geobacillus	M	2.606929			
	RB40	M	2.723305			
	Unclassified RB40 genus	M	2.73251			
	Thermus	M	2.724025			
**Age group**	Nocardioidaceae	Senior	2.544902	Marasmius	Adult	2.82278
	Oxalobacteraceae	Senior	2.991608	Agaricales_family_incertae_sedis	Adult	2.83465
	Ralstonia	Senior	2.988875	Auriculariaceae	Adult	3.40098
	N09	Senior	2.481695	Auricularia	Adult	3.406538
	Planctomycetes	Senior	2.635051	Auriculariales	Adult	3.476242
				Clavicipitaceae	Senior	3.262227
				unclassified_Clavicipitaceae_genus	Senior	3.270884
				Eurotiales	Senior	4.265596
				Trichocomaceae	Senior	4.265596
