# Supplementary material for: The feline cutaneous and oral microbiota are influenced by breed and environment
Source: PLoS One. 2019 Jul 30;14(7):e0220463. doi: 10.1371/journal.pone.0220463 (PMC6667137; doi:10.1371/journal.pone.0220463)
Supplement: S7 Table — (DOCX) [file pone.0220463.s014.docx]

**Table S7. Taxa determined to be differentially abundant on the skin across hair length groups with LEfSe (LDA>2.5, p<0.01).**

|  | **Bacteria** | | | **Fungi** | | |
| --- | --- | --- | --- | --- | --- | --- |
|  | **Taxa** | **Group** | **LDA score** | **Taxa** | **Group** | **LDA score** |
| **Dorsum** | Rhodospirillales | Very short | 3.151268 | Unclassified Pleosporaceae genus | Very short | 3.839302 |
|  | Anaerolineae | Medium | 2.939502 | Pleosporaceae | Very short | 4.076013 |
| **Ear canal** | Other Oxalobacteraceae | Long | 2.913865 |  |  |  |
| **Nostril** | Other Lactobacillales genus | Medium | 3.317047 |  |  |  |
|  | Other Lactobacillales | Medium | 3.301913 |  |  |  |
| **Oral cavity** | Unclassified Clostridiales family | Medium | 2.879556 | Unclassified Sordariomycetes family | Medium | 2.523213 |
|  | Unclassified Clostridiales genus | Medium | 2.885784 | Unclassified Sordariomycetes order | Medium | 2.981653 |
|  | Fusibacter | Long | 3.063423 | Unclassified Sordariomycetes genus | Medium | 3.057213 |
|  | Acidaminobacteraceae | Long | 3.085213 | Mycosphaerellaceae | Short | 3.400643 |
